# Effects of Plant Polysaccharides on Meat Quality of Squabs Based on Ileal Metabolomics

**DOI:** 10.3390/life16050705

**Published:** 2026-04-22

**Authors:** Jie Ren, Jiajia Liu, Huiguo Yang, Haiying Li, Xiaoyu Zhao, Yafei Liang, Mingcong Ding, Yuanhao Li, Haiying He, Xiaobin Li

**Affiliations:** 1 College of Animal Science, Xinjiang Agricultural University, Urumqi 830052, China; 15133391369@163.com (J.R.); 13629439845@163.com (H.L.); vz_zxy@163.com (X.Z.); m13934057294@163.com (Y.L.); 15329625726@163.com (M.D.); 17769614800@163.com (Y.L.); 2 Moyu Blue Sea Pigeon Industry Co., Ltd., Hetian 848100, China; 13964992580@163.com; 3 Institute of Animal Husbandry, Xinjiang Academy of Animal Husbandry, Urumqi 830011, China; 13809951421@163.com (H.Y.); 15132311203@163.com (H.H.)

**Keywords:** astragalus polysaccharide, glycyrrhiza polysaccharide, meat quality, slaughter performance, metabolomics of ileal contents

## Abstract

Plant polysaccharides, such as Astragalus polysaccharide (APS) and Glycyrrhiza polysaccharide (GPS), hold potential as feed additives, yet their individual and synergistic effects on squab meat quality remain unclear. In this study, 192 healthy, 15-day-old, early-weaned Silver King squabs were assigned to one of four dietary treatments for 28 days: a control group (CK), an APS group, a GPS group, and a combined APS + GPS group (AG). Slaughter traits, organ indices, liver antioxidant capacity, and meat quality were evaluated across the four groups. Results indicated that supplementation with APS, GPS, and AG enhanced several slaughter traits compared to CK, including live weight, carcass weight, full-eviscerated weight, half-eviscerated weight, and leg muscle weight. GPS and AG supplementation improved color parameters in both breast and leg muscles, with AG showing the most favorable tenderness-related outcomes. Additionally, AG supplementation enhanced liver antioxidant capacity, as evidenced by increased total antioxidant capacity and superoxide dismutase activity. Given AG’s superior overall performance, the ileal metabolomics analysis focused on comparing CK and AG. Metabolomics data revealed clear group separation and significant changes in amino acid-related pathways. In summary, while APS and GPS individually improved certain traits, their combined supplementation yielded the most favorable results, likely through enhanced antioxidant capacity and altered ileal amino acid metabolism.

## 1. Introduction

Meat quality is a complex trait influenced by various factors, including genetics and nutrition. Understanding the biochemical pathways and metabolic processes that affect meat quality is critical for enhancing production efficiency and meeting consumer demand. The ileum, integral to nutrient absorption and microbial fermentation, plays a critical role in the overall metabolism of meat-producing animals. Metabolomics serves as a powerful tool for investigating the relationship between metabolic processes and complex traits, such as meat quality. Pigeon meat, known for its high-quality protein, unsaturated fatty acids, and various minerals, is low in fat and offers tender, flavorful meat. This makes it a popular high-protein, low-fat poultry product among consumers, with meat quality directly impacting both market value and breeding profitability [1]. In large-scale breeding operations, stressors like early weaning and high stocking density may compromise intestinal barrier function and reduce the antioxidant capacity of meat pigeons, leading to poorer meat quality, including decreased muscle water retention, reduced tenderness, and insufficient flavor compound accumulation [2,3]. These factors hinder the high-quality development of the pigeon breeding industry. Therefore, the search for green, safe, and efficient feed additives to address these issues is essential for the sustainable development of poultry farming.

As a natural, green feed additive, plant polysaccharides offer advantages such as the absence of toxic side effects, no residue, and versatile applications, making them widely used in livestock and poultry farming [4,5]. Among these, Astragalus polysaccharide (APS) and Glycyrrhiza polysaccharide (GPS), as two typical green and safe plant polysaccharides, have demonstrated promising results in improving livestock and poultry production due to their remarkable effects. Research indicates that APS enhances the physical and chemical properties of muscle by activating the antioxidant enzyme system and modulating the muscle’s energy metabolism pathways [6], while GPS promotes the deposition of flavor amino acids and fatty acids in muscle by regulating gut microbiota and facilitating nutrient digestion and absorption [7]. Numerous studies have explored the effects of APS and GPS on the meat quality of broilers, pigs, and other livestock and poultry species [8,9], but their influence on key quality traits such as water-holding capacity, tenderness, and the color parameters L*, a*, and b* in squab muscle remains unclear. Therefore, the present study aimed to compare the effects of dietary APS, GPS, and their combined supplementation on slaughter traits, organ indices, meat quality, and liver antioxidant capacity in squabs. Given the ileum’s critical role in nutrient absorption and host–microbiota interactions, ileal metabolomics offers valuable insights into the intestinal metabolic alterations associated with polysaccharide supplementation and meat quality formation. Consequently, ileal metabolomics was employed to investigate the intestinal metabolic basis underlying the optimal treatment response. This study design enabled the evaluation of both individual and combined effects of APS and GPS on squab meat quality and related physiological traits. To our knowledge, few studies have simultaneously assessed the individual and combined impacts of APS and GPS on squab meat quality and linked these phenotypic changes to ileal metabolomic alterations.

## 2. Materials and Methods

### 2.1. Ethical Considerations

All animal care and experimental procedures adhered to the Guidelines for the Care and Use of Experimental Animals in China and were approved by the Experimental Animal Ethics Committee of the Institute of Animal Husbandry, Academy of Animal Husbandry, Xinjiang Uygur Autonomous Region (project license number: 202602).

### 2.2. Additive

APS and GPS used in the study were sourced from Lyman Xiangrong Biotechnology Co., Ltd. The purities of APS and GPS were 68% and 40%, respectively. The control group (CK) received a basal diet without polysaccharide supplementation; the APS group received the basal diet supplemented with 800 mg/kg APS; the GPS group received the basal diet supplemented with 450 mg/kg GPS; and the combined group (AG) received the basal diet supplemented with both 800 mg/kg APS and 450 mg/kg GPS. These supplementation levels were based on the manufacturer’s recommendations and previous studies.

### 2.3. Animal and Experimental Design

The animal trial was conducted at Moyu Blue Sea Pigeon Industry Development Co., Ltd. (Hotan, Xinjiang, China). A total of 192 healthy 15-day-old early-weaned Silver King squabs were randomly assigned to four dietary treatments: a control group (CK), an APS group, a GPS group, and a combined APS + GPS group (AG), with 12 replicates per treatment and 4 squabs per replicate, resulting in 48 squabs per treatment. The experiment lasted for 28 days. Squabs were housed in cages (45 cm × 50 cm × 60 cm) with ad libitum access to feed and water. The lighting schedule was 16 h of light per day, and room temperature was maintained at 15 ± 5 °C. All groups were managed under identical environmental and feeding conditions, with the only variation being the dietary supplementation strategy. The replicate cage was considered the experimental unit for production-related measurements, while the individual bird served as the experimental unit for slaughter traits, organ indices, meat quality, antioxidant indices, and metabolomics analysis. During the experiment, all squabs were fed a basal diet formulated to meet the nutritional requirements of meat pigeons. The composition and nutrient levels of the basal diet are shown in Table 1.

### 2.4. Sample Collection

At the end of the experiment, one squab was randomly selected from each replicate, yielding 12 squabs per treatment group for sample collection. After euthanasia, the heart, lung, liver, gizzard, glandular stomach, spleen, abdominal fat, bursa of Fabricius, and kidneys were collected for the determination of slaughter traits and organ indices. Breast and leg muscle samples were collected for meat quality analysis. Liver tissues were immediately frozen in liquid nitrogen and stored at −80 °C for antioxidant analysis. Ileal contents were collected under sterile conditions, frozen in liquid nitrogen, and stored at −80 °C for metabolomics analysis.

#### 2.4.1. Antioxidant Capacity

Total antioxidant capacity (T-AOC), total superoxide dismutase (T-SOD) activity, glutathione peroxidase (GSH-Px) activity, and malondialdehyde (MDA) content in liver tissue were quantified using commercial assay kits following the manufacturer’s instructions (Nanjing Jiancheng Bioengineering Institute, Nanjing, China).

#### 2.4.2. Determination of Slaughter Performance

Live weight, slaughter weight, half-eviscerated weight, full-eviscerated weight, breast muscle weight, leg muscle weight, abdominal fat weight, and organ weights (heart, lung, liver, gizzard, glandular stomach, spleen, bursa of Fabricius, and kidney) were measured. The slaughter rate, half-eviscerated rate, and full-eviscerated rate were calculated based on the method outlined by Ye et al. [10].

#### 2.4.3. Routine Meat Quality Determination

Post-slaughter, ipsilateral breast and leg muscles were collected for the determination of meat color, pH value, shear force, and water loss rate, following Ncho’s method [11]. Meat color parameters, including lightness (L*), redness (a*), and yellowness (b*), were assessed at 45 min postmortem on the surface of the breast and leg muscles using a 3nh NR110 portable precision colorimeter (Shenzhen Threenh Technology Co., Ltd., Shenzhen, China), with a 4 mm measuring aperture and d/8° optical geometry, in accordance with CIE standards. The instrument was calibrated using a standard white tile before measurement. For the leg muscle, measurements were taken from the same anatomical region of each sample on a relatively uniform muscle surface, avoiding visible fat, fascia, connective tissue, and damaged areas. The measuring aperture was positioned perpendicular to the muscle surface, with three measurements taken at different points and averaged. Muscle pH was measured in the corresponding region using a portable pH meter (pH-Star, Matthäus, Germany), with three replicate measurements recorded and averaged for statistical analysis. Prior to measurement, the pH meter was calibrated using standard buffer solutions as per the manufacturer’s guidelines. Drip loss was determined by weighing standardized muscle samples collected from the same anatomical region. After removing visible fat and connective tissue, the samples were stored under controlled conditions for a fixed period, then removed, gently blotted dry, and reweighed. Drip loss was expressed as the percentage of weight loss relative to the initial sample weight. Shear force was measured by treating muscle samples in a constant-temperature water bath (DK-S24, Shanghai Jinghong Experimental Equipment Co., Ltd., Shanghai, China) under standardized conditions and then assessing tenderness using a digital tenderness meter (C-LM3B, Tenovo, Beijing, China). Each parameter was measured in triplicate, and the average value was used for statistical analysis.

### 2.5. Metabolomics Analysis

Ileal contents were selected for metabolomic analysis due to the ileum’s key role in nutrient absorption and host–microbiota metabolic interactions, where dietary plant polysaccharides may exert early effects on intestinal metabolism. Based on the overall phenotypic evaluation across the four groups, the combined APS + GPS treatment (AG) demonstrated the most favorable response. Consequently, the main manuscript focuses on comparing the CK and AG groups in ileal metabolomics analysis to investigate the metabolic basis underlying the optimal treatment effect. The metabolomic results for the APS and GPS groups are included in the Supplementary Materials.

A 100 mg sample of ileal content was ground in liquid nitrogen and transferred to an EP tube, followed by the addition of 500 μL of 80% methanol aqueous solution. The sample was vortexed, placed in an ice bath for 5 min, and centrifuged at 15,000× *g* at 4 °C for 20 min. A portion of the supernatant was diluted with mass spectrometry-grade water to reduce the methanol content to 53%, followed by a second centrifugation at 15,000× *g* at 4 °C for 20 min. The final supernatant was collected and injected into the LC–MS system for analysis (Q Exactive HF/Q Exactive HF-X/Orbitrap Explorer IS 120/Orbitrap Explorer IS 480, Thermo Fisher, Bremen, Germany). LC–MS/MS analysis was performed by Novogene Co., Ltd. (Beijing, China).

Chromatographic separation was carried out using a Hypesil Gold C18 column (100 × 2.1 mm, 1.9 μm; Thermo Fisher, Waltham, MA, USA). The sample injection volume was 3 μL, the column temperature was set to 40 °C, and the flow rate was 0.2 mL/min. In positive ion mode (POS), the mobile phases consisted of 0.1% formic acid in water (A) and methanol (B). In negative ion mode (NEG), mobile phase A was 90% acetonitrile with 5 mM ammonium acetate, and mobile phase B was 50% acetonitrile with 5 mM ammonium acetate. The gradient elution program is shown in Table 2.

Electrospray ionization (ESI) was employed in both POS and NEG, with the scanning range set from *m*/*z* 100 to 1500. The ESI conditions were as follows: spray voltage, 3.5 kV; sheath gas flow rate, 35 psi; auxiliary gas flow rate, 10 L/min; capillary temperature, 320 °C; S-lens RF level, 60; and auxiliary gas heater temperature, 350 °C. Secondary MS/MS scanning was conducted using data-dependent acquisition.

Quality control of QC samples and principal component analysis (PCA) of all samples were performed using R software (R-3.4.3). PCA and partial least squares discriminant analysis (PLS-DA) were carried out using metaX for metabolomics data processing. Differential metabolites were identified based on the criteria of VIP > 1, *p* < 0.05, and fold change (FC) ≥ 1.2 or ≤0.833. Volcano plots and bubble plots were generated using ggplot2 in R (v4.2.1). The KEGG database was used for functional annotation and pathway enrichment analysis of metabolites, with pathways showing *p* < 0.05 considered significantly enriched.

For quality control, pooled QC samples were prepared by mixing equal aliquots from all experimental samples, and blank samples were included to eliminate background ions. Raw LC–MS data were converted to mzXML format using ProteoWizard and processed with XCMS for peak extraction, quantification, and alignment. Metabolite annotation was conducted by matching accurate mass, adduct information, and MS/MS spectral data against the Novogene in-house high-quality secondary spectral database (NovoMetDB) with a mass tolerance of 10 ppm. The original quantitative data were normalized according to the platform workflow to obtain relative peak areas, and compounds with a CV > 30% in QC samples were excluded from further analysis.

### 2.6. Statistical Analysis

Data were analyzed using SPSS 27.0 software (IBM Corp., Armonk, NY, USA) and are presented as mean ± SD. The replicate cage was considered the experimental unit for production-related traits, while the individual bird was the experimental unit for slaughter performance, organ indices, meat quality, antioxidant indices, and metabolomics analysis. Phenotypic data across the four groups were analyzed using one-way analysis of variance (ANOVA). When a significant treatment effect was detected, multiple comparisons among means were performed using Duncan’s multiple range test. For metabolomics analysis in the main manuscript, statistical comparisons focused on the CK and AG groups. Pearson correlation analysis was performed to assess relationships between differential metabolites and meat quality traits. A *p*-value of <0.05 was considered statistically significant, and *p* < 0.01 was considered highly significant. Figures were generated using GraphPad Prism 8.0.2 and ChiPlot (https://www.chiplot.online/).

## 3. Results

### 3.1. Effects of Dietary Plant Polysaccharides on Slaughter Performance of Squabs

As shown in Table 3, dietary plant polysaccharide supplementation significantly influenced several slaughter-performance traits in squabs. Compared to the CK group, live weight, carcass weight, full-eviscerated weight, half-eviscerated weight, and leg muscle weight were significantly increased in the APS, GPS, and AG groups (*p* < 0.01). Breast muscle weight was significantly higher in the AG group compared to the CK group (*p* < 0.05), while the APS and GPS groups showed intermediate values and did not significantly differ from either CK or AG. However, carcass rate, full-eviscerated rate, half-eviscerated rate, breast muscle rate, and leg muscle rate were not significantly affected by the dietary treatments (*p* > 0.05). Overall, both APS and GPS supplementation independently improved major slaughter traits, with the combined treatment also yielding a favorable overall response.

### 3.2. Effects of Dietary Plant Polysaccharides on Meat Quality of Squabs

#### 3.2.1. Breast Muscle Quality

As shown in Table 4, dietary plant polysaccharide supplementation significantly influenced several breast muscle quality traits. The pH value of breast muscle was significantly lower in the GPS and AG groups compared to the CK and APS groups (*p* < 0.01). The L* value tended to decrease with supplementation and was significantly lower in the AG group than in the CK group (*p* < 0.05), while the APS and GPS groups showed intermediate values. In contrast, both a* and b* values increased after supplementation. These parameters were significantly higher in the GPS and AG groups compared to the CK and APS groups (*p* < 0.01), with the AG group exhibiting the highest mean values. No significant differences in water loss rate were observed among treatments (*p* > 0.05). Shear force was lowest in the AG group and significantly lower than in the APS group (*p* < 0.05), while the CK and GPS groups displayed intermediate values. These results suggest that GPS and AG primarily affected breast muscle color traits, with AG also associated with lower shear force.

#### 3.2.2. Leg Muscle Quality

As shown in Table 5, significant differences in several leg muscle quality parameters were observed among treatments. The pH value was significantly lower in the GPS group than in both the CK and APS groups (*p* < 0.01), with the AG group showing an intermediate value. The L* value decreased following supplementation. Compared to the CK group, the APS group showed a significant decrease (*p* < 0.05), whereas the GPS and AG groups exhibited highly significant reductions (*p* < 0.01). In contrast, a* and b* values increased after supplementation, with the GPS and AG groups showing significantly higher values than the CK and APS groups (*p* < 0.01). No significant difference in water loss rate was found among the four groups (*p* > 0.05). Shear force was lowest in the AG group, significantly lower than in the CK and APS groups (*p* < 0.05), while the GPS group showed an intermediate response. Overall, both GPS and AG improved leg muscle color, with the AG group demonstrating the best tenderness-related performance.

### 3.3. Effects of Dietary Plant Polysaccharides on Organ Indices of Squabs

As shown in Table 6, dietary plant polysaccharide supplementation had limited effects on most organ indices. The heart index in the GPS group was significantly higher than in the CK group (*p* < 0.01), while the APS and AG groups showed intermediate values. The lung index in the GPS group was also significantly higher than in the CK group (*p* < 0.05), with no significant differences observed between the APS and AG groups and the CK group. No significant differences were found among treatments for liver, spleen, glandular stomach, gizzard, kidney, or bursa of Fabricius indices (*p* > 0.05). These results suggest that dietary APS and GPS supplementation had relatively minor effects on the relative weights of most visceral organs under the experimental conditions.

### 3.4. Effects of Dietary Plant Polysaccharides on Liver Antioxidant Capacity in Squabs

As shown in Table 7, dietary polysaccharide supplementation significantly influenced liver antioxidant indices. T-AOC increased after supplementation, with the AG group showing a significantly higher value than the CK group (*p* < 0.01). The APS and GPS groups exhibited intermediate values, with GPS being numerically higher than APS. T-SOD activity was highest in the AG group and significantly higher than in the GPS group (*p* < 0.01). The CK and APS groups displayed intermediate values with no significant differences from either the AG or GPS groups. GSH-Px activity varied among treatments, with the APS group showing significantly higher levels than the GPS group (*p* < 0.05), while the CK and AG groups showed intermediate values. For MDA, the GPS group exhibited significantly higher levels than the CK group (*p* < 0.01), with the AG group showing an intermediate value and no significant difference from the CK group. Overall, the combined AG treatment demonstrated the most favorable antioxidant profile, particularly in terms of T-AOC and T-SOD activity.

### 3.5. Effects of Dietary Plant Polysaccharides on Ileal Metabolomics in Squabs

#### 3.5.1. Sample Quality Control (QC) Analysis

**Figure 1:**
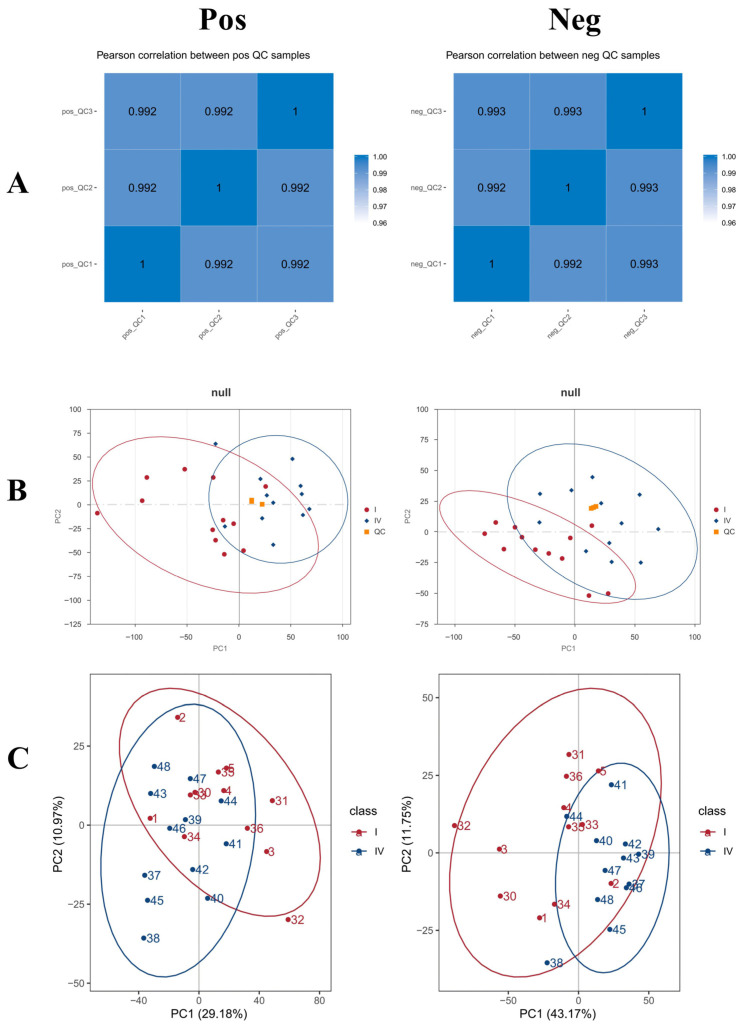
Metabolomics characteristics of each sample. (**A**) Correlation analysis of QC samples. (**B**) Principal component analysis (PCA) of the total sample. (**C**) Principal component analysis (PCA). The left side is positive ion mode (Pos) and the right side is negative ion mode (Neg).

As the AG group exhibited the best overall phenotypic performance among the four dietary treatments, subsequent ileal metabolomics analysis in the main text focused on the comparison between the CK and AG groups. The metabolomic results for the APS and GPS groups are provided in the Supplementary Materials. Pearson correlation coefficients between QC samples were calculated based on the relative quantitative values of metabolites, with all correlation coefficients greater than 0.99, indicating stable detection and high data quality (Figure 1A). The peaks extracted from all test and QC samples were analyzed by PCA, showing that QC sample points clustered together, further demonstrating the stability and high quality of the metabolite identification process (Figure 1B).

#### 3.5.2. Principal Component Analysis

PCA was performed to observe the general distribution trends between the two sample groups. The PCA results in both NEG and POS revealed that sample points from the CK group (I) and AG group (IV) showed a distinct clustering trend, with clear separation between groups. In NEG, the separation was explained by PC1 (43.17%) and PC2 (11.75%), while in POS, the separation was explained by PC1 (29.18%) and PC2 (10.97%) (Figure 1C).

#### 3.5.3. PLS-DA

**Figure 2:**
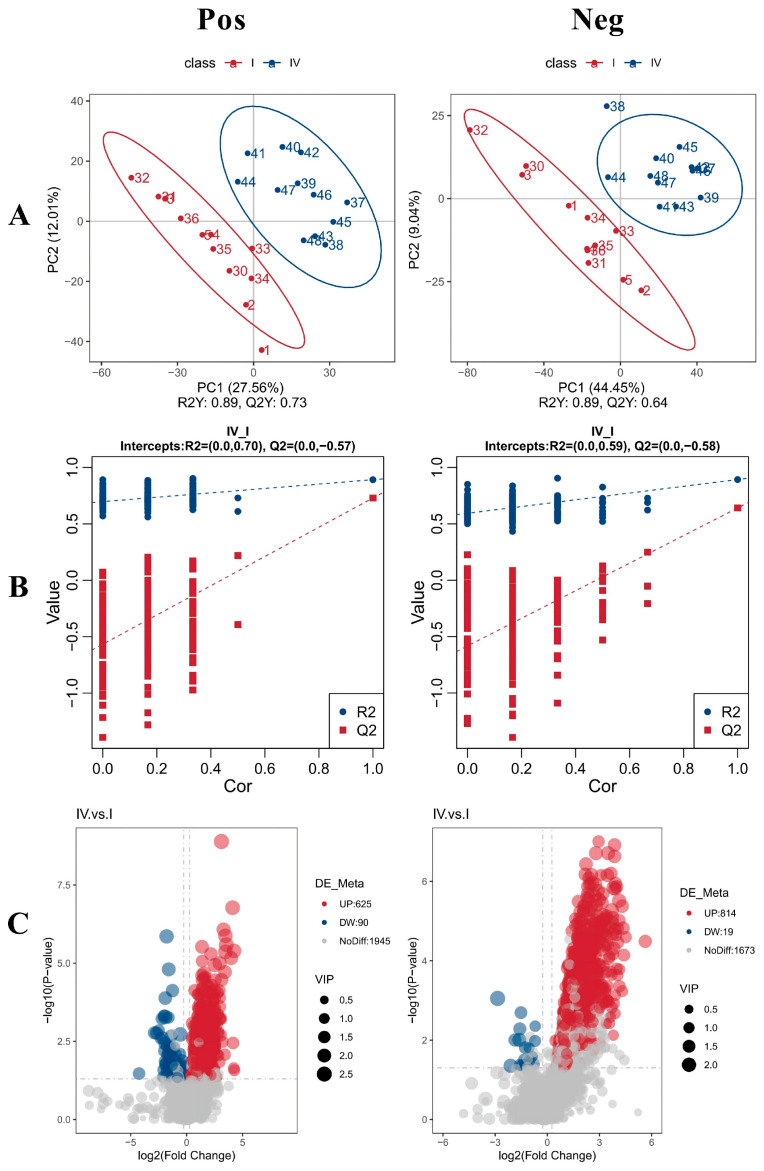
Metabolomics characteristics of each sample. (**A**) PLS scores of intestinal contents in CK group and AG group. (**B**) PLS-DA analysis model replacement test chart. (**C**) volcano map. The left side is positive ion mode (Pos) and the right side is negative ion mode (Neg).

To assess the quality of the model, model sorting and verification are often conducted to check for potential “over-fitting.” The presence of “over-fitting” reflects the accuracy of model construction. If the model is not over-fitted, it indicates that the model adequately represents the sample, providing a solid foundation for identifying the biomarker group. If the model is over-fitted, it suggests that the model is unsuitable for describing the sample, making further analysis invalid. The specific method involves randomly scrambling the grouping labels of each sample before modeling and forecasting. For each model, corresponding R^2^ and Q^2^ values are calculated, and their regression lines are generated by scrambling and modeling the data 200 times. When R^2^ is greater than Q^2^ and the intercept of the Q^2^ regression line with the Y-axis is below 0, it indicates that the model is not over-fitted. In this study, the PLS-DA positive ion model had R^2^Y = 0.89 and Q^2^Y = 0.73, while the PLS-DA negative ion model had R^2^Y = 0.89 and Q^2^Y = 0.64. Both R^2^Y and Q^2^Y exceeded 0.5, and R^2^Y was close to 1, demonstrating that the model possesses high explanatory power and predictive ability, and is both stable and reliable. In both ion modes, the data from the CK and AG groups were well-separated, effectively distinguishing between the two sample groups (Figure 2A). The Q^2^ values of the positive and negative ion models were −0.57 and −0.58, respectively. The intercepts of the Q^2^ regression lines with the Y-axis were below 0, and R^2^ was greater than Q^2^, confirming that the model is not over-fitted and is capable of accurately representing the sample. This ensures the model is suitable for subsequent differential metabolite screening (Figure 2B).

#### 3.5.4. Volcanic Diagram and Matchstick Diagram of Differential Metabolites

Using non-targeted metabolomics (LC-MS/MS), 1945 metabolites in POS and 1673 metabolites in NEG were detected. Among these, 715 metabolites in POS showed significant differences, with 625 up-regulated and 90 down-regulated. In NEG, 833 metabolites were significantly different, with 814 up-regulated and 19 down-regulated (Figure 2C).

#### 3.5.5. Differential Metabolite Chord Diagram

**Figure 3:**
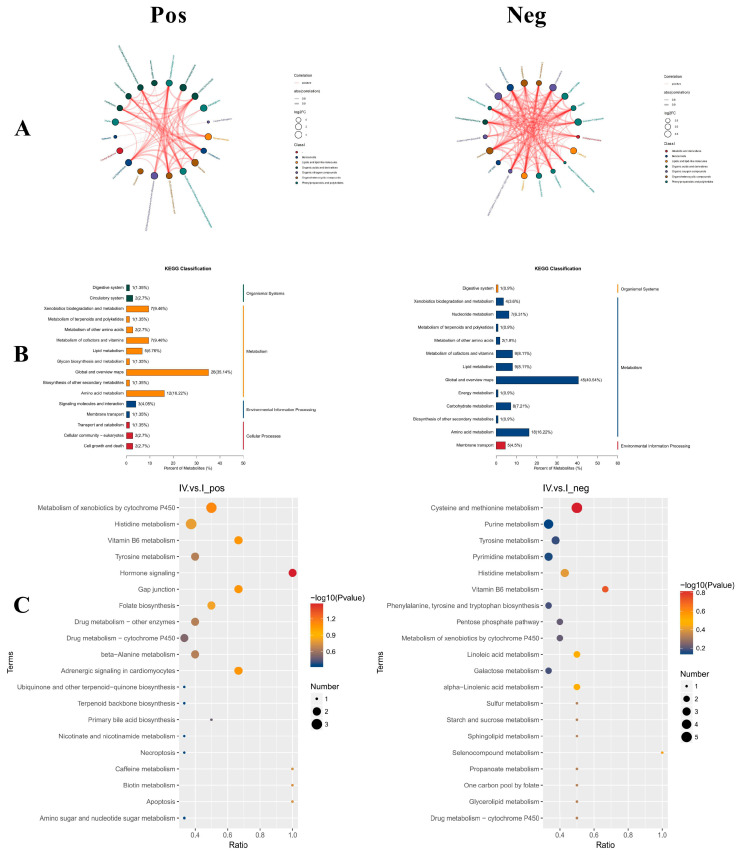
Analysis results of intestinal differential metabolites between CK group and AG group. (**A**) Chord diagram. (**B**) KEGG classification diagram of differential metabolites. (**C**) Bubble diagram of pathway enrichment analysis of differential metabolites. The greater the Ratio value, the higher the enrichment degree of differential metabolites in this pathway, the color of the point represents the *p*-value of the hypergeometric test, and the size of the point represents the number of differential metabolites in the corresponding pathway. The left side is positive ion mode (Pos) and the right side is negative ion mode (Neg).

The chord diagram is a visual tool that illustrates the correlation between data. Nodes are arranged radially along the circumference, with chord connections of varying widths representing the degree of correlation between nodes. By drawing a chord diagram based on the correlation coefficient of different metabolites, the interactions and relationships between metabolites in the sample are reflected. The top 20 differential metabolites, ordered by ascending *p*-values, were selected for the chord diagram in Figure 3A. The results revealed that in POS, the interactions related to phospholipids were more prominent, while in NEG, the co-occurrence relationships centered on organic acids were dominant.

#### 3.5.6. KEGG Classification and Enrichment Analysis

Based on the classified data of metabolites in the KEGG database, statistical analysis was conducted on the annotated differential metabolites (Figure 3B). In POS, the differential metabolites were categorized into four groups: Organismal Systems, Metabolism, Environmental Information Processing, and Cellular Processes. In NEG, the differential metabolites were divided into three categories: Organismal Systems, Metabolism, and Environmental Information Processing. Notably, metabolic pathways represented the largest proportion in both POS and NEG.

To further elucidate the potential functions of the annotated differential metabolites in the AG and CK groups, KEGG functional enrichment analysis was performed (Figure 3C), showing the top 20 enriched KEGG metabolic pathways. In POS, the differential metabolites were primarily enriched in exogenous metabolism, histidine metabolism, vitamin B6 metabolism, tyrosine metabolism, and hormone signaling pathways mediated by cytochrome P450, with the hormone signaling pathway showing the most significant differences. In NEG, the differential metabolites were mainly concentrated in cysteine and methionine metabolism, purine metabolism, pyrimidine metabolism, histidine metabolism, and vitamin B6 metabolism, with cysteine and methionine metabolism showing the most significant changes. The pathways shared between the two modes include tyrosine metabolism, histidine metabolism, vitamin B6 metabolism, and cytochrome P450-mediated xenobiotic metabolism.

#### 3.5.7. Screening of Marker Metabolites

**Figure 4:**
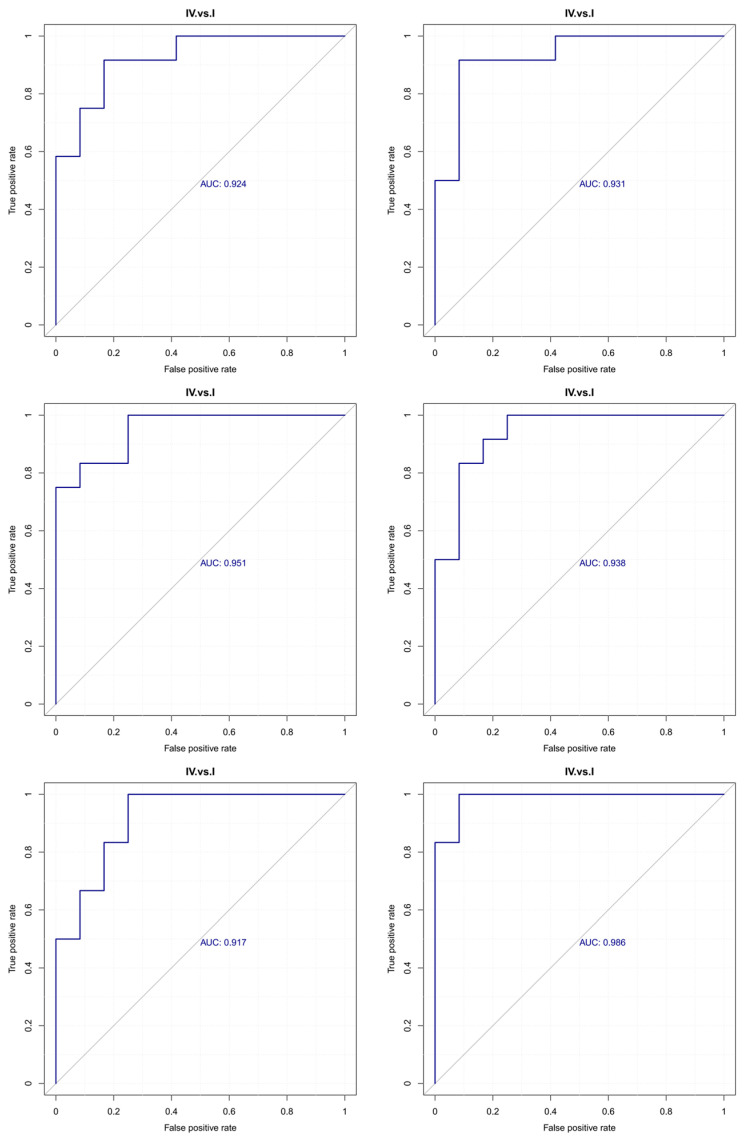
ROC curve of differential metabolites. The blue diagonal line represents the reference line (AUC = 0.5), indicating the performance of random classification.

The area under the ROC curve (AUC) is used to evaluate the sensitivity and specificity of biomarkers in predicting event occurrence. The sensitivity and specificity of each metabolite are determined by the optimal threshold of the ROC curve. When AUC = 0.5, the biomarker has no predictive value, indicating no effect on event prediction. AUC values greater than 0.5 indicate predictive ability, with values closer to 1 representing higher prediction accuracy. Generally, AUC values between 0.5 and 0.7 suggest low prediction accuracy, values between 0.7 and 0.9 indicate moderate prediction accuracy, and values above 0.9 indicate high prediction accuracy. Notably, the following differential metabolites exhibited excellent predictive performance: Aspartyl-Arginine (AUC = 0.986), Aspartyl-Lysine (AUC = 0.951), Gamma-Glutamylserine (AUC = 0.924), Threonyl-Lysine (AUC = 0.938), and N(6)-Glycyl-L-Lysine (GLY-GLY-ILE, AUC = 0.917). With AUC values exceeding 0.80, these metabolites demonstrated strong predictive capabilities and can be considered potential marker metabolites, as shown in Figure 4.

### 3.6. Correlation Analysis

**Figure 5:**
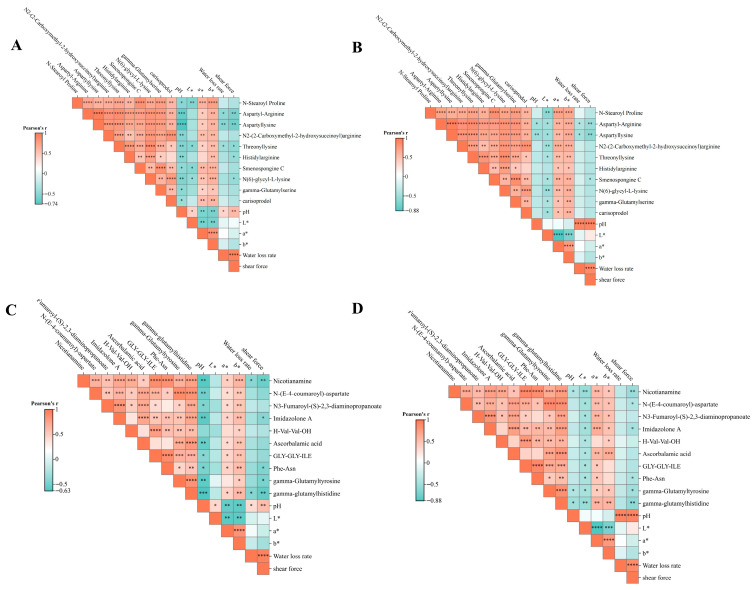
Correlation Analysis: (**A**) Correlation of breast muscle quality under positive ions. (**B**) Correlation of leg muscle quality under positive ions. (**C**) Correlation of breast muscle quality under negative ions. (**D**) Correlation of leg muscle quality under negative ions. Red means positive correlation and blue means negative correlation. * *p* < 0.05, ** *p* < 0.01, *** *p* < 0.001, **** *p* < 0.0001 versus the CK group.

The top 10 key differential metabolites (VIP > 1.0, FC > 1.2 or FC < 0.833, *p*-value < 0.05) were selected for correlation analysis with meat quality traits, as shown in Figure 5. In POS, a* and b* values of both breast and leg muscles were positively correlated with N-Stearoyl Proline, Aspartyl-Arginine, Aspartyl-Lysine, N2-(2-carboxymethyl-2-hydroxysuccinoyl) Arginine, Threonyl-Lysine, Histidyl-Arginine, Smenospongine C, N(6)-Glycyl-L-Lysine, Gamma-Glutamylserine, and Carisoprodol. Conversely, pH, shear force, and water binding capacity of both muscle types were negatively correlated with several metabolites, particularly Aspartyl-Arginine, Aspartyl-Lysine, Threonyl-Lysine, Histidyl-Arginine, and Smenospongine C. In NEG, a* and b* values of both breast and leg muscles were positively correlated with Nicotianamine, N-(E-4-coumaroyl)-Aspartate, N3-Fumaroyl-(S)-2,3-diaminopropane, Imidazolone A, H-Val-Val-OH, Ascorbalamic acid, GLY-GLY-ILE, Phe-Asn, Gamma-Glutamyltyrosine, and Gamma-Glutamylhistidine. Meanwhile, pH, shear force, and water retention in both muscle types showed negative correlations with certain metabolites, especially Nicotianamine, N-(E-4-coumaroyl)-Aspartate, Imidazolone A, Phe-Asn, Gamma-Glutamyltyrosine, and Gamma-Glutamylhistidine.

## 4. Discussion

In this study, both APS and GPS individually improved certain phenotypic traits, while the combined APS + GPS treatment generally yielded the most favorable overall response. These results indicate that each additive contributed to the observed effects, with the combined treatment proving more effective in enhancing the overall phenotypic profile under the experimental conditions. Based on these findings, the main text’s metabolomics analysis focused on comparing the CK and AG groups to explore the intestinal metabolic basis underlying the optimal treatment response. Meat quality is typically assessed through pH, color, water-holding capacity, and tenderness, which collectively influence consumer acceptance and market value [12]. Lower shear force and drip loss, alongside improved redness, are generally regarded as indicators of superior meat quality [13,14,15]. In this study, dietary plant polysaccharide supplementation reduced shear force and improved color parameters in both breast and leg muscles, reflecting an overall enhancement in muscle quality. These changes may be partly attributed to the increased antioxidant capacity observed in the AG group. Oxidative stress accelerates lipid peroxidation and protein oxidation, compromising muscle cell membrane integrity, postmortem protein stability, and water-holding capacity [16,17]. Therefore, the observed increases in T-AOC and T-SOD activities may have contributed to the improved tenderness and meat color. In addition to antioxidant regulation, intestinal metabolic alterations likely played a role in the observed improvements in meat quality. Untargeted metabolomics revealed that differential metabolites between the CK and AG groups were predominantly enriched in amino acid-related pathways, such as cysteine and methionine metabolism, histidine metabolism, vitamin B6 metabolism, and tyrosine metabolism. These pathways are closely linked to protein turnover, redox balance, and energy metabolism [18]. Previous studies have shown that polysaccharide supplementation can modulate intestinal microbiota and host metabolism, thereby influencing nutrient digestion, amino acid utilization, and muscle deposition [4,6,7]. Consequently, the present results suggest that combined APS and GPS supplementation is associated with enhanced meat quality and altered ileal metabolic profiles. Among the identified metabolites, Aspartyl-Arginine, Aspartyl-Lysine, Gamma-Glutamylserine, Threonyl-Lysine, N(6)-Glycyl-L-Lysine, and GLY-GLY-ILE exhibited relatively high predictive performance in ROC analysis and were significantly associated with meat quality traits. These metabolites are primarily involved in small-peptide metabolism and amino acid transport, suggesting a potential link between ileal amino acid-related metabolism and muscle quality traits. Notably, several of these metabolites were positively correlated with a* and b* values and negatively correlated with pH and shear force, indicating their potential as candidate biomarkers for favorable color and tenderness traits. However, these relationships should be interpreted with caution, as the study provides statistical associations rather than direct mechanistic evidence.

Aspartyl-Arginine may be involved in metabolic processes related to energy metabolism and redox balance [19]. However, its specific role in regulating squab meat quality was not directly tested in this study and therefore remains speculative. Additionally, asparagine-related metabolism has been linked to energy supply, which could be relevant to postmortem muscle physiology. However, its direct relationship with calcium homeostasis and shear force was not experimentally verified in this study [20]. Intestinal amino acid metabolism is also strongly influenced by the gut microbiota, with microbiota-mediated regulation of amino acid utilization potentially affecting host nutrient homeostasis and muscle quality formation [21]. Aspartyl-Lysine, another peptide-related metabolite, may reflect differences in peptide turnover or protein metabolism, potentially influencing postmortem physicochemical traits such as tenderness, water retention, and flavor [22,23]. Gamma-Glutamylserine could also be related to glutamyl peptide turnover and subsequent glutamate-related metabolic processes, which are associated with muscle pH stability, protein denaturation, and water-holding capacity [24,25,26]. Furthermore, since myoglobin oxidation state plays a critical role in meat color, metabolites involved in amino acid and peptide metabolism may indirectly influence the conversion between oxymyoglobin and metmyoglobin, thus contributing to variations in a* and b* values [27]. Threonyl-Lysine, N(6)-Glycyl-L-Lysine, and GLY-GLY-ILE were also significantly associated with multiple meat quality traits in this study. Previous research has shown that threonine and lysine nutrition affects collagen synthesis, protein deposition, antioxidant status, and muscle quality characteristics [28,29], while lysine-related regulation may also impact myofibrillar protein properties and water retention [16,30]. Moreover, short peptide-related metabolites like GLY-GLY-ILE may reflect differences in peptide metabolism linked to tenderness-related properties [31,32]. The significant up-regulation of these metabolites observed in this study may suggest that alterations in ileal amino acid and small-peptide metabolism were associated with improved meat color traits and reduced shear force. However, these findings should be interpreted cautiously, as the study provides correlation-based evidence rather than establishing direct causal relationships between specific metabolites and meat quality traits.

Notably, the differential pathways identified were predominantly enriched in amino acid metabolism rather than lipid metabolism. This suggests that the beneficial effects of plant polysaccharides in this study may be more closely related to the regulation of nitrogen metabolism, peptide turnover, and redox balance in the intestine. As the ileum is a critical site for nutrient absorption and microbial metabolite interactions, alterations in ileal metabolites may reflect upstream regulatory events influencing muscle development and meat quality [4,18]. Combined with the correlation analysis, these results support the idea that intestinal metabolic remodeling may contribute to the improvement of squab meat quality. However, the precise biological roles of these metabolites in regulating meat quality remain to be fully elucidated. In conclusion, the findings of this study suggest that dietary supplementation with plant polysaccharides is associated with improved meat quality in squabs, potentially through enhanced antioxidant capacity and alterations in ileal amino acid metabolism. The identified metabolites offer valuable insights into the intestine–muscle axis and may serve as potential biomarkers for evaluating meat quality in poultry.

Slaughter performance is a key economic trait used to evaluate poultry production, serving as a critical index for assessing meat production efficiency and the effectiveness of feeding strategies [33]. This set of indicators provides an intuitive reflection of nutrient deposition across different tissues or sections of the same tissue in meat pigeons. Similar to other poultry species, meat pigeons concentrate muscle mass primarily in the breast and legs, and their yield and quality directly influence overall meat production performance [34]. Core indicators, such as slaughter rate, half-eviscerated rate, breast muscle rate, and leg muscle rate, are essential for measuring meat performance. Research by Qiao et al. found that supplementing AA male broilers with low and high-dose compound polysaccharide diets led to a significant increase in slaughter rate, breast muscle rate, and leg muscle rate in the high-dose group, while the breast muscle rate in the low-dose group increased by 2.4% [6]. Liu et al. reported that the full clean bore rate and half clean bore rate were significantly improved in groups T5, T6, and T7, with the slaughter rate of groups T6 and T7 notably higher than that of the control group (T1). Additionally, the breast muscle rate and leg muscle rate in the FAP supplementation groups were significantly higher than those in the control group [35]. In the present study, dietary supplementation with a combination of APS and GPS significantly improved carcass weight, full-eviscerated weight, half-eviscerated weight, and leg muscle weight in the AG group compared to the control group. These results are consistent with previous findings and suggest that APS enhances antioxidant capacity, reducing muscle oxidative damage, while GPS improves intestinal barrier function and increases the efficiency of amino acid and energy utilization. The synergistic effect of these two polysaccharides may optimize nutrient distribution to muscle tissues, thereby improving slaughter performance.

Organ weight and index serve as indicators of organ growth and development, reflecting the body’s metabolic, digestive, absorptive, and immune functions [36]. Immune organs such as the bursa of Fabricius and spleen play a pivotal role in poultry growth. Wei et al. demonstrated that supplementation of APS in varying concentrations significantly influenced the immune organ indices of 7-day-old broilers, suggesting APS enhances the development of internal organs in the early stages of broiler growth [37]. In the current experiment, the addition of a combined polysaccharide resulted in higher bursa of Fabricius weight and index in the AG group compared to the CK group, while spleen weight and index were lower in the AG group. This could be attributed to the spleen being a peripheral immune organ with less constant immune stimulation. Additionally, the relatively limited nutritional allocation to the spleen may explain its less pronounced development. Digestive organs are vital for nutrient digestion and absorption [38]. Generally, a larger digestive organ index indicates more efficient nutrient digestion and absorption [39]. The present study showed that dietary polysaccharide supplementation increased heart, gizzard, glandular stomach, and lung weights while significantly reducing liver index. These findings suggest that polysaccharide supplementation promotes the development of the heart, gizzard, and lung, enhancing immune performance, while inhibiting liver development. This inhibition may be linked to elevated levels of aspartate aminotransferase and alanine aminotransferase, though further investigation is required to clarify the underlying mechanisms.

T-AOC provides a comprehensive measure of the body’s antioxidant status, while SOD is a critical antioxidant enzyme that protects cells from oxidative damage. Dietary supplementation with APS has been shown to significantly increase serum SOD levels in broilers [40]. Additionally, GPS was found to notably enhance T-AOC in broiler livers, indicating its potent role in improving liver antioxidant capacity [41]. Furthermore, Wang’s study on fermented Astragalus membranaceus demonstrated significant improvements in serum T-SOD and T-AOC activities in weaned piglets upon dietary supplementation [42]. Similarly, Jin observed that varying concentrations of APS led to significant increases in SOD, GSH-Px, CAT, and GR activities, while also reducing MDA levels in the serum of normal Roman chicks [43]. These findings are consistent with the present study, where both Astragalus and GPS enhanced SOD and T-AOC activities in the liver of weaned pigeons. However, an increase in MDA levels in the experimental group compared to the control warrants further investigation. Possible explanations include insufficient adaptation time for the body to respond to the polysaccharides, as well as suboptimal sample storage conditions, such as excessive temperature or prolonged storage, which could lead to MDA degradation. Despite this, the overall results suggest that dietary supplementation with APS and GPS can effectively enhance the antioxidant capacity of young pigeons, strengthening the liver’s antioxidant system by boosting T-AOC levels and SOD activity. This, in turn, provides robust support for liver health.

In this study, dietary supplementation with APS and GPS resulted in improved meat quality, slaughter performance, and hepatic antioxidant capacity in squabs. These findings align with previous research indicating that plant-derived polysaccharides can enhance growth performance, antioxidant status, and product quality in poultry through the improvement of intestinal health, nutrient utilization, and metabolic homeostasis [4,5,6,7]. However, a limitation of this study must be acknowledged: only a single supplementation level was tested, selected based on the manufacturer’s recommendation and prior studies. Consequently, the dose–response relationship and the optimal supplementation level could not be determined. Further research using graded doses is necessary to clarify the dose-dependent effects of plant polysaccharide supplementation.

## 5. Conclusions

Unlike previous studies that primarily focused on single polysaccharide supplementation or conventional phenotypic outcomes, this study expands current knowledge by assessing both the individual and combined effects of APS and GPS in squabs. Additionally, it integrates phenotypic analysis with ileal metabolomic profiling and metabolite–trait association analysis. These findings offer valuable insights into the intestinal metabolic basis underlying meat quality improvements in squabs. In conclusion, dietary supplementation with combined APS and GPS enhanced slaughter performance, antioxidant status, and meat quality in squabs. These improvements were associated with alterations in ileal metabolite profiles, particularly in amino acid-related pathways. Several differential metabolites were significantly correlated with meat quality traits and may serve as potential biomarkers. However, further experimental validation is needed to elucidate the underlying mechanisms.

## Figures and Tables

**Table 1:** Composition and nutrient levels of raw grain and pellets in the basal diet (air-dried basis) %.

Ingredients	Contents
Corn	52.00
Soybean meal	24.50
Soybean oil	1.00
Pea	10.50
Wheat	4.00
Yeast powder	2.00
Stone powder	0.80
Glucose	0.50
Baking soda	0.40
NaCl	0.40
CaHPO_4_	0.60
L-Lys·HCl	0.24
DL-Met	0.16
Mycotoxin adsorbent	0.10
Premix ^1^	1.80
Wheat bran	1.00
Total	100.00
Nutrient components	
ME/(MJ/kg)	11.90
CP	18.14
Ca	1.73
P	0.57
Lys	0.77
Met	0.36

Note: ^1^ Premixed feed supplies per kilogram of feed: VA 27,000 IU, VD_3_ 90,000 IU, VE 850 mg, VK_3_ 92 mg, VB_2_ 160 mg, VB_6_ 115 mg, Nicotinamide 1050 mg, calcium D-pantothenate 520 mg, D-biotin 2 mg, folic acid) 20 mg, Cu 340 mg, Fe 1700 mg, Mn 1700 mg, Zn 1400 mg; nutrient composition is actually measured value and metabolic energy is calculated value.

**Table 2:** Gradient elution program of Chromatographic.

Time/min	Mobile Phase A/%	BMobile Phase B/%
0	98	2
1.5	98	2
3	15	85
10	0	100
10.1	98	2
11	98	2
12	98	2

**Table 3:** Effects of dietary APS, GPS, and their combination on slaughter performance of squabs.

Item	CK	APS	GPS	AG
Live weight	458.75 ± 25.69 ^Bb^	500.33 ± 15.34 ^Aa^	499.75 ± 31.00 ^Aa^	503.08 ± 17.85 ^Aa^
Carcass weight	413.33 ± 28.30 ^Bb^	455.48 ± 19.27 ^Aa^	449.66 ± 31.16 ^Aa^	453.18 ± 20.20 ^Aa^
Full-eviscerated weight	342.44 ± 23.68 ^Bb^	384.31 ± 20.90 ^Aa^	379.84 ± 30.63 ^Aa^	378.05 ± 16.47 ^Aa^
Half-eviscerated weight	382.73 ± 25.16 ^Bb^	421.59 ± 21.06 ^Aa^	418.33 ± 32.53 ^Aa^	423.28 ± 15.19 ^Aa^
Breast muscle weight	91.15 ± 12.88 ^b^	100.20 ± 8.81 ^ab^	99.42 ± 7.92 ^ab^	100.87 ± 12.52 ^a^
Leg muscle weight	22.09 ± 1.28 ^Bb^	24.73 ± 1.74 ^Aa^	24.98 ± 2.39 ^Aa^	25.28 ± 2.95 ^Aa^
Carcass rate	90.05 ± 1.70	91.02 ± 1.92	89.96 ± 1.96	90.06 ± 1.76
Full-eviscerated rate	74.63 ± 2.63	76.78 ± 2.63	75.96 ± 2.49	75.16 ± 2.32
Half-eviscerated rate	83.42 ± 2.76	84.23 ± 2.28	83.66 ± 2.54	84.14 ± 0.71
Breast muscle rate	26.54 ± 2.49	26.13 ± 2.54	26.20 ± 1.34	26.64 ± 2.69
Leg muscle rate	6.46 ± 0.30	6.45 ± 0.50	6.59 ± 0.60	6.68 ± 0.60

Note: CK, control group; APS, Astragalus polysaccharide group; GPS, Glycyrrhiza polysaccharide group; AG, combined APS + GPS group. Within the same row, values with different lowercase letters indicate a significant difference (*p* < 0.05), and values with different uppercase letters indicate a highly significant difference (*p* < 0.01).

**Table 4:** Effects of dietary APS, GPS, and their combination on breast muscle quality of squabs.

Breast Muscle	CK	APS	GPS	AG
pH	6.63 ± 0.15 ^Aa^	6.66 ± 0.16 ^Aa^	6.37 ± 0.19 ^Bb^	6.35 ± 0.12 ^Bb^
L*	28.11 ± 1.80 ^Aa^	27.56 ± 1.46 ^Aab^	26.21 ± 2.02 ^ABbc^	25.45 ± 1.52 ^Bc^
a*	16.65 ± 3.06 ^Bc^	18.10 ± 1.89 ^Bc^	21.28 ± 3.15 ^Ab^	23.59 ± 2.29 ^Aa^
b*	9.23 ± 1.67 ^Bc^	10.49 ± 2.08 ^Bc^	13.62 ± 1.79 ^Ab^	15.20 ± 1.21 ^Aa^
Water loss rate/%	8.65 ± 2.69	7.69 ± 2.67	7.30 ± 4.11	7.15 ± 3.22
Shear force/N	29.04 ± 5.98 ^AaBb^	33.50 ± 9.94 ^Aa^	27.81 ± 10.08 ^AaBb^	22.78 ± 7.24 ^Bb^

Note: CK, control group; APS, Astragalus polysaccharide group; GPS, Glycyrrhiza polysaccharide group; AG, combined APS + GPS group. Within the same row, values with different lowercase letters indicate a significant difference (*p* < 0.05), and values with different uppercase letters indicate a highly significant difference (*p* < 0.01).

**Table 5:** Effects of dietary APS, GPS, and their combination on leg muscle quality of squabs.

Leg Muscle	CK	APS	GPS	AG
pH	6.57 ± 0.17 ^AaBb^	6.60 ± 0.16 ^Aa^	6.42 ± 0.11 ^Bc^	6.47 ± 0.11 ^ABbc^
L*	39.64 ± 3.95 ^Aa^	35.29 ± 1.97 ^Bb^	32.81 ± 2.81 ^Bc^	32.20 ± 2.00 ^Bc^
a*	11.54 ± 2.22 ^Bb^	12.65 ± 2.15 ^Bb^	18.86 ± 2.25 ^Aa^	17.85 ± 2.97 ^Aa^
b*	11.66 ± 1.79 ^Bb^	12.19 ± 1.51 ^Bb^	15.31 ± 1.79 ^Aa^	15.30 ± 1.54 ^Aa^
Water loss rate/%	7.90 ± 3.96	8.73 ± 3.80	6.43 ± 2.28	6.41 ± 2.48
Shear force/N	33.03 ± 9.44 ^a^	32.60 ± 9.12 ^a^	26.27 ± 7.79 ^ab^	24.17 ± 5.26 ^b^

Note: CK, control group; APS, Astragalus polysaccharide group; GPS, Glycyrrhiza polysaccharide group; AG, combined APS + GPS group. Within the same row, values with different lowercase letters indicate a significant difference (*p* < 0.05), and values with different uppercase letters indicate a highly significant difference (*p* < 0.01).

**Table 6:** Effects of dietary APS, GPS, and their combination on organ indices of squabs (g).

Item	CK	APS	GPS	AG
Heart weight	5.24 ± 0.69 ^Bb^	5.69 ± 0.75 ^AaBb^	6.09 ± 0.95 ^Aa^	5.46 ± 0.27 ^ABb^
Liver weight	12.53 ± 2.43	11.45 ± 1.32	11.73 ± 2.15	11.20 ± 2.03
Spleen weight	0.79 ± 0.44	0.65 ± 0.24	0.79 ± 0.33	0.60 ± 0.35
Glandular stomach weight	1.26 ± 0.23	1.29 ± 0.23	1.18 ± 0.35	1.37 ± 0.20
Gizzard weight	8.50 ± 1.42	9.47 ± 0.96	9.39 ± 1.21	8.52 ± 1.01
Kidney weight	3.24 ± 1.21	3.55 ± 1.06	3.51 ± 1.32	3.03 ± 0.96
Bursa of Fabricius weight	0.78 ± 0.32	0.94 ± 0.21	0.80 ± 0.31	0.93 ± 0.32
Lung weight	6.09 ± 1.52 ^b^	7.06 ± 1.79 ^ab^	7.92 ± 2.18 ^a^	6.58 ± 1.65 ^ab^
Abdominal fat weight	4.33 ± 2.26	4.90 ± 1.94	5.23 ± 2.03	3.87 ± 1.62

Note: CK, control group; APS, Astragalus polysaccharide group; GPS, Glycyrrhiza polysaccharide group; AG, combined APS + GPS group. Within the same row, values with different lowercase letters indicate a significant difference (*p* < 0.05), and values with different uppercase letters indicate a highly significant difference (*p* < 0.01).

**Table 7:** Effects of dietary APS, GPS, and their combination on liver antioxidant indices of squabs.

Item	CK	APS	GPS	AG
T-AOC/(U/mL)	0.15 ± 0.02 ^Bc^	0.16 ± 0.03 ^ABbc^	0.17 ± 0.02 ^Aab^	0.18 ± 0.02 ^Aa^
T-SOD/(U/mL)	673.80 ± 48.49 ^ABb^	665.61 ± 59.80 ^ABb^	651.79 ± 36.20 ^Bb^	721.10 ± 48.28 ^Aa^
GSH-Px/(U/mL)	124.69 ± 17.07 ^ab^	130.61 ± 12.19 ^a^	113.66 ± 14.07 ^b^	124.95 ± 20.92 ^ab^
MDA/(nmol/mL)	0.39 ± 0.09 ^Bb^	0.41 ± 0.12 ^Bb^	0.53 ± 0.09 ^Aa^	0.44 ± 0.08 ^ABb^

Note: CK, control group; APS, Astragalus polysaccharide group; GPS, Glycyrrhiza polysaccharide group; AG, combined APS + GPS group. Within the same row, values with different lowercase letters indicate a significant difference (*p* < 0.05), and values with different uppercase letters indicate a highly significant difference (*p* < 0.01).

## Data Availability

The data that support the findings of this study are available from the corresponding author upon reasonable request.

## Supplementary Materials

The following supporting information can be downloaded at: https://www.mdpi.com/article/10.3390/life16050705/s1.

## Abbreviations

The following abbreviations are used in this manuscript:

APS	Astragalus polysaccharides
GPS	Glycyrrhiza polysaccharides
CK	control group
AG	Astragalus polysaccharides and Glycyrrhiza polysaccharides supplementation group
T-AOC	total antioxidant capacity
T-SOD	total superoxide dismutase
GSH-Px	glutathione peroxidase
MDA	malondialdehyde
LC-MS	liquid chromatography–mass spectrometry
LC-MS/MS	liquid chromatography–tandem mass spectrometry
ESI	electrospray ionization
m/z	mass-to-charge ratio
QC	quality control
PCA	principal component analysis
PLS-DA	partial least squares discriminant analysis
VIP	variable importance in projection
FC	fold change
KEGG	Kyoto Encyclopedia of Genes and Genomes
ROC	receiver operating characteristic
AUC	area under the curve
SPSS	Statistical Package for the Social Sciences
SD	standard deviation
