# Supplementary figures and images for: Effects of Plant Polysaccharides on Meat Quality of Squabs Based on Ileal Metabolomics
Source: Life (Basel). 2026 Apr 22;16(5):705. doi: 10.3390/life16050705 (PMC13208362; doi:10.3390/life16050705)

# II.vs.I\_neg

Terms

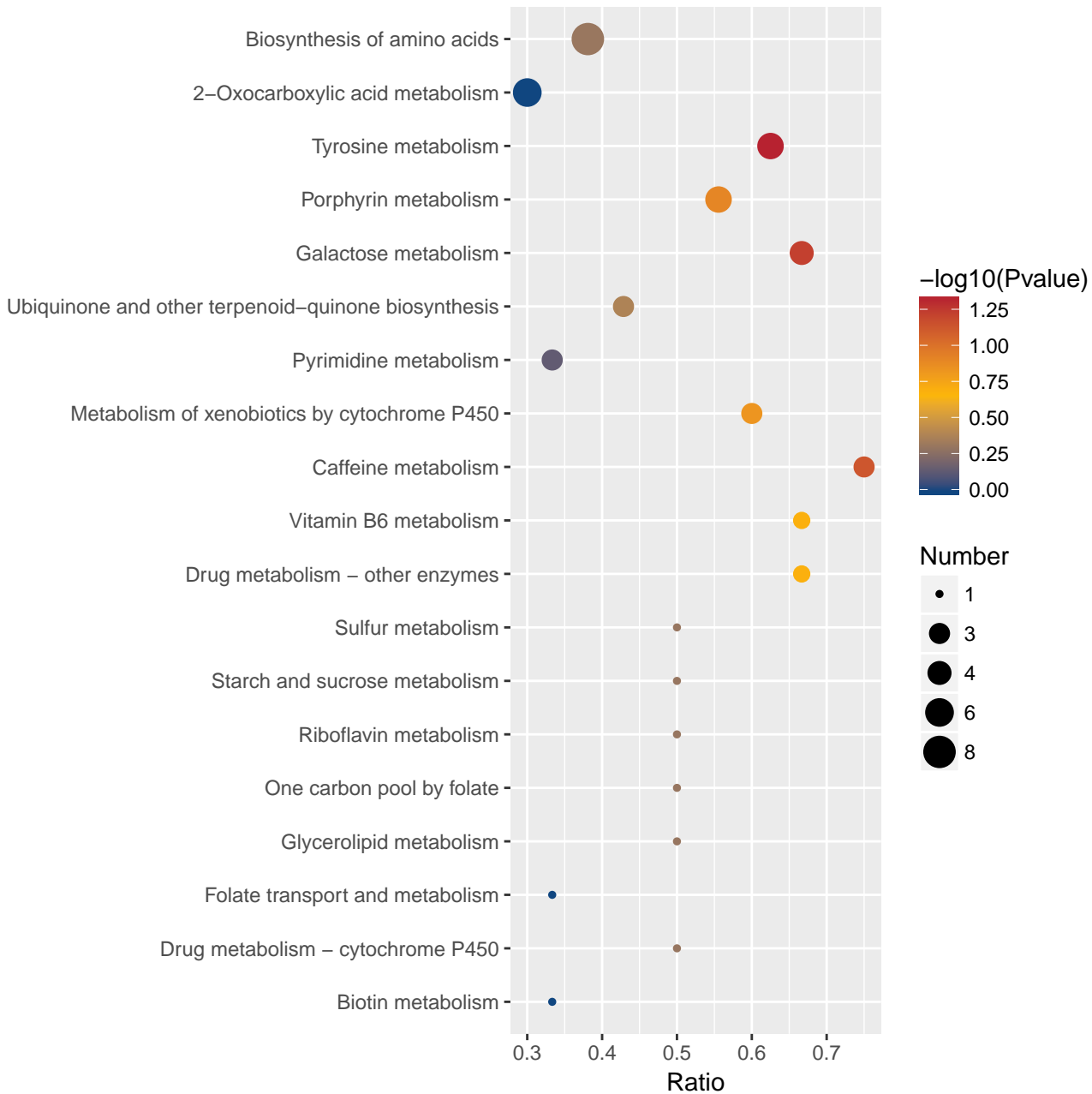

Supplement: Supplementary file 1 [file life-16-00705-s001.zip › APS,GPS/II.vs.I_neg.KEGG_Enrich.scatterplot.pdf]

II.vs.I

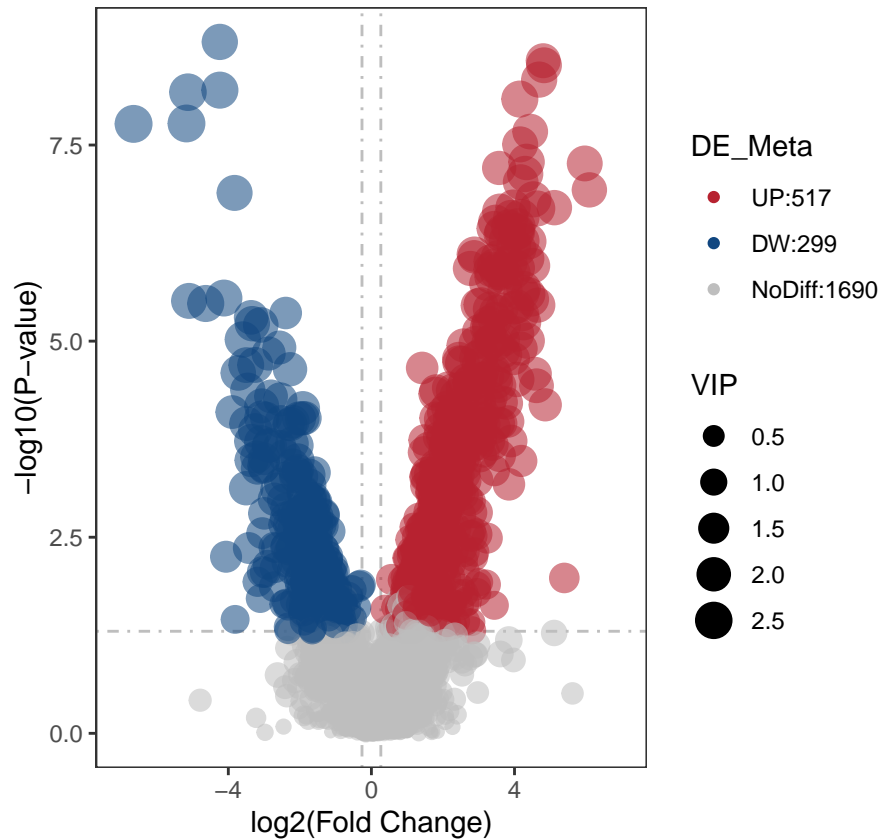

Supplement: Supplementary file 1 [file life-16-00705-s001.zip › APS,GPS/II.vs.I_neg.xls.volcano.pdf]

KEGG Classification

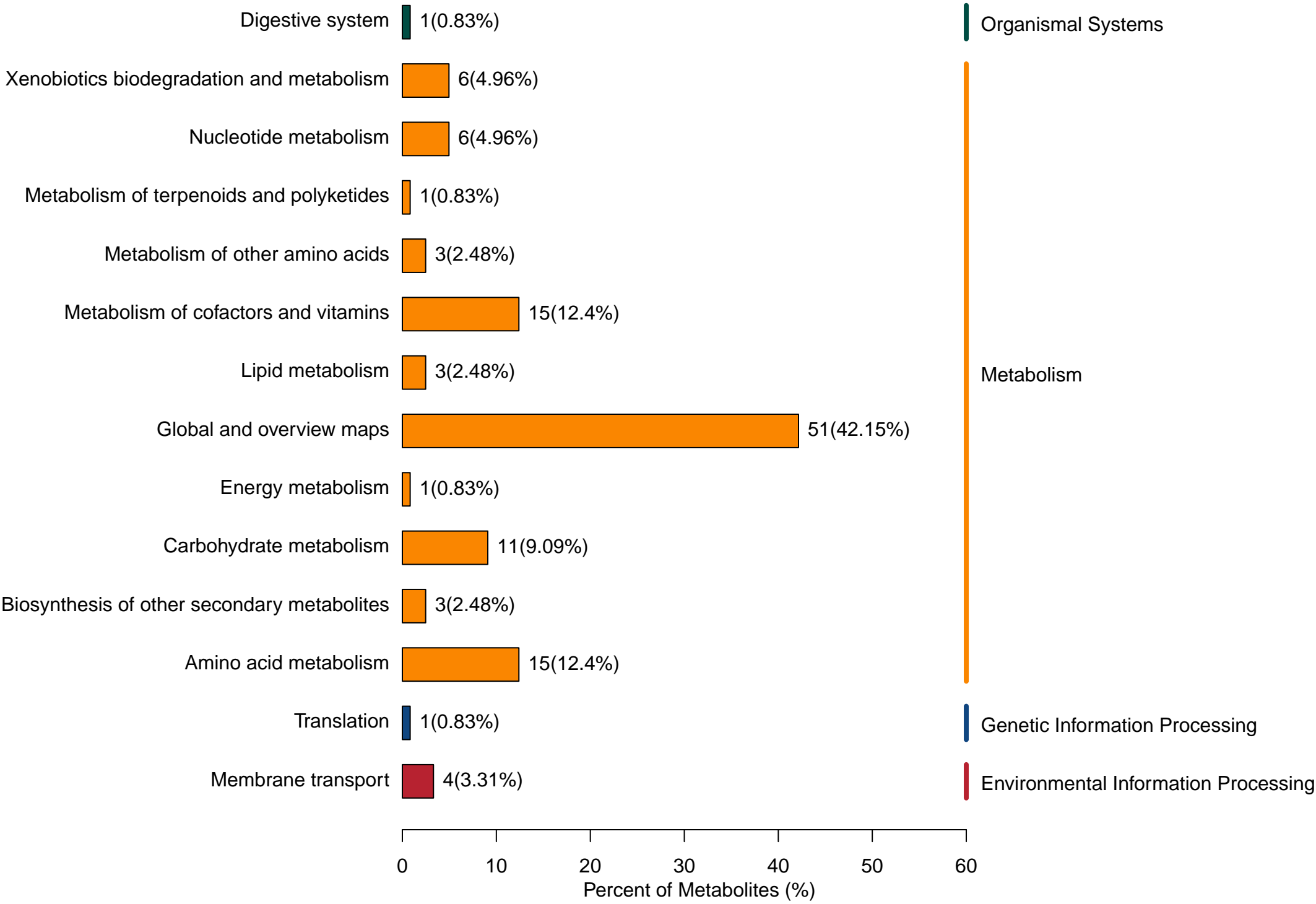

Supplement: Supplementary file 1 [file life-16-00705-s001.zip › APS,GPS/II.vs.I_neg_KEGG_classification.pdf]

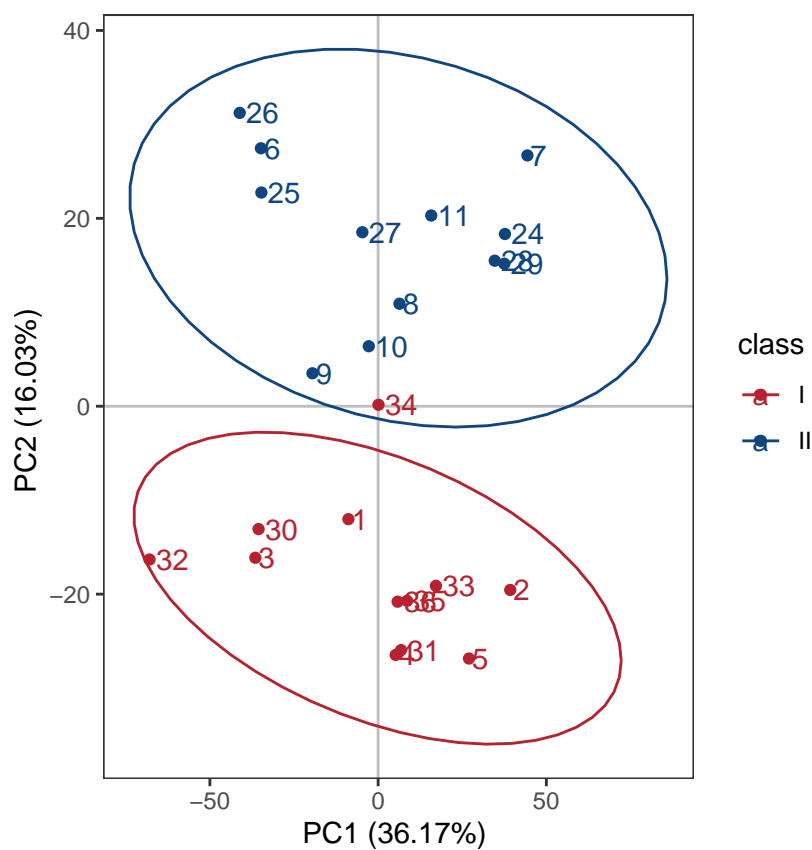

Supplement: Supplementary file 1 [file life-16-00705-s001.zip › APS,GPS/II.vs.I_neg_PCA.pdf]

class I II

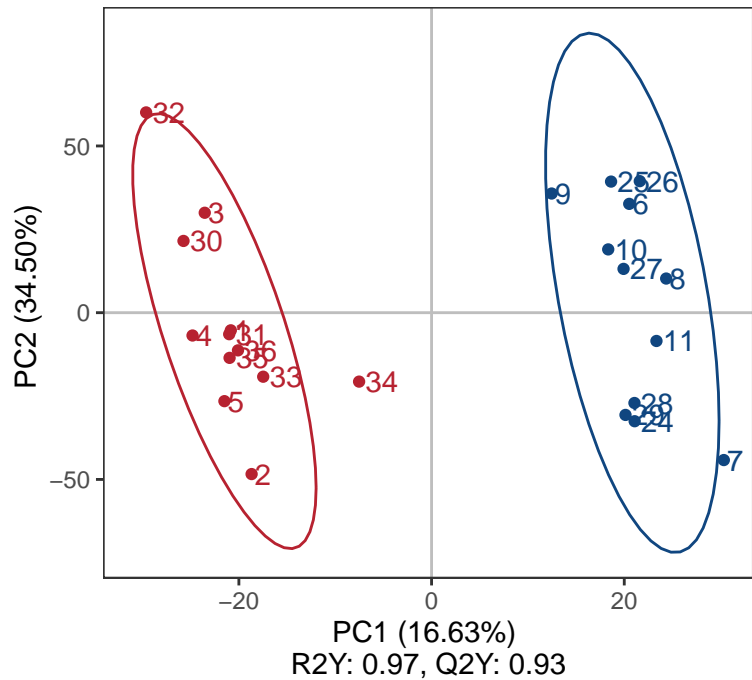

Supplement: Supplementary file 1 [file life-16-00705-s001.zip › APS,GPS/II.vs.I_neg_PLSDA-score.pdf]

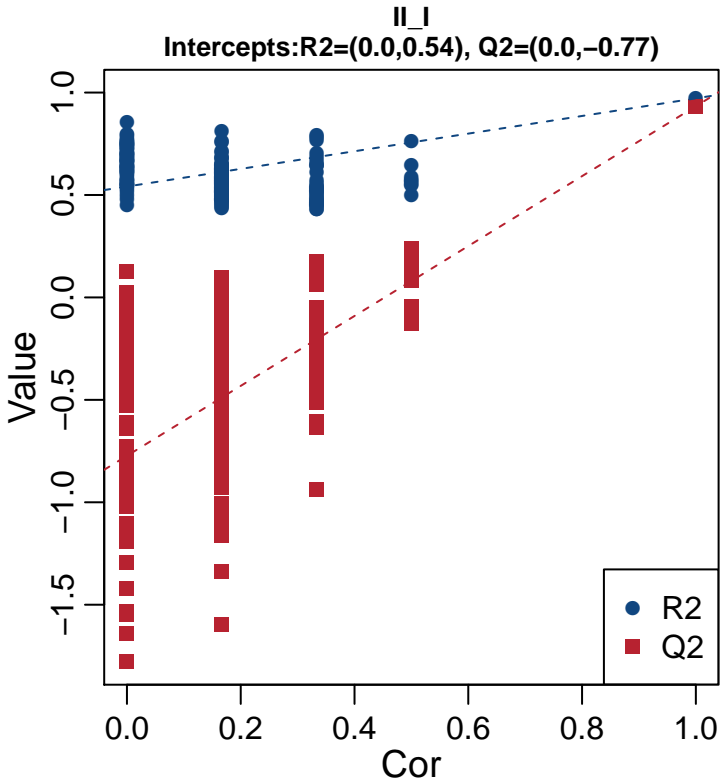

Supplement: Supplementary file 1 [file life-16-00705-s001.zip › APS,GPS/II.vs.I_neg_PLSDA-valid.pdf]

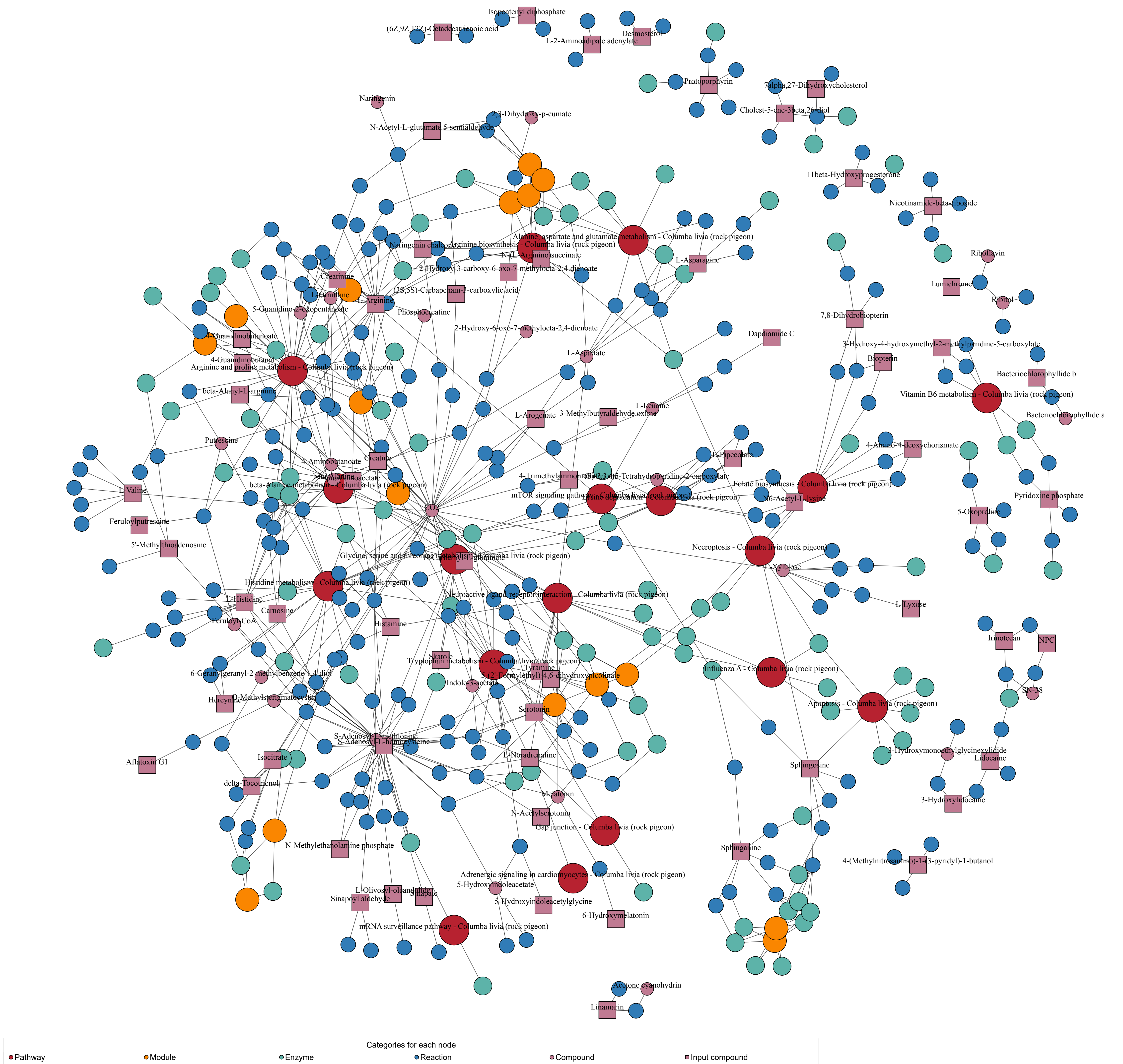

Supplement: Supplementary file 1 [file life-16-00705-s001.zip › APS,GPS/II.vs.I_pos.KEGG_net.pdf]

II.vs.I

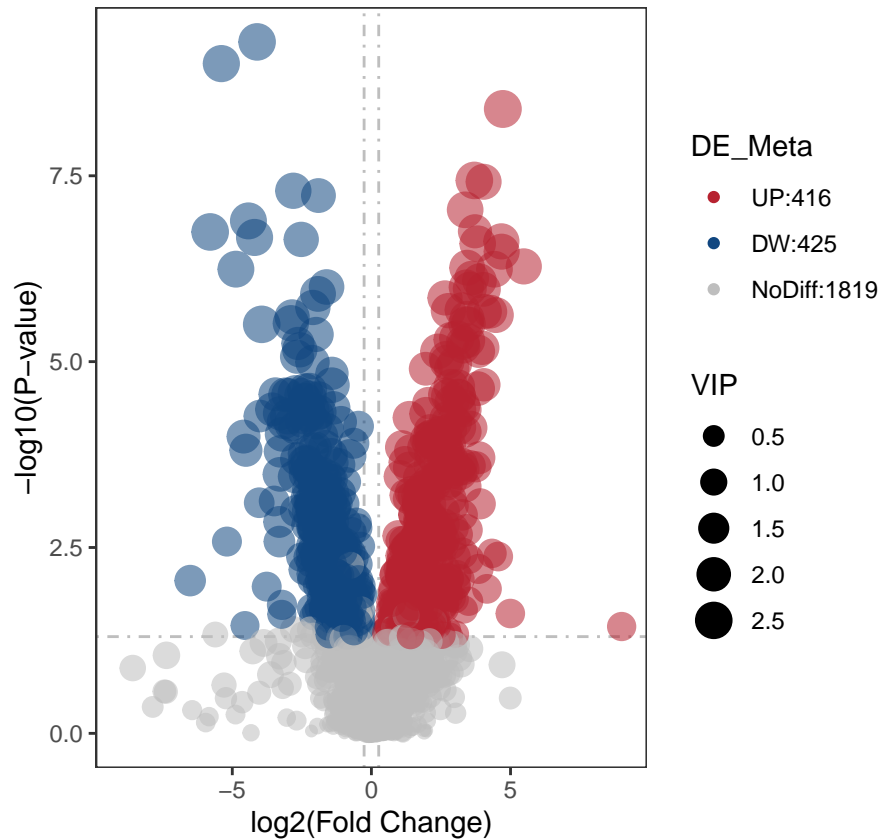

Supplement: Supplementary file 1 [file life-16-00705-s001.zip › APS,GPS/II.vs.I_pos.xls.volcano.pdf]

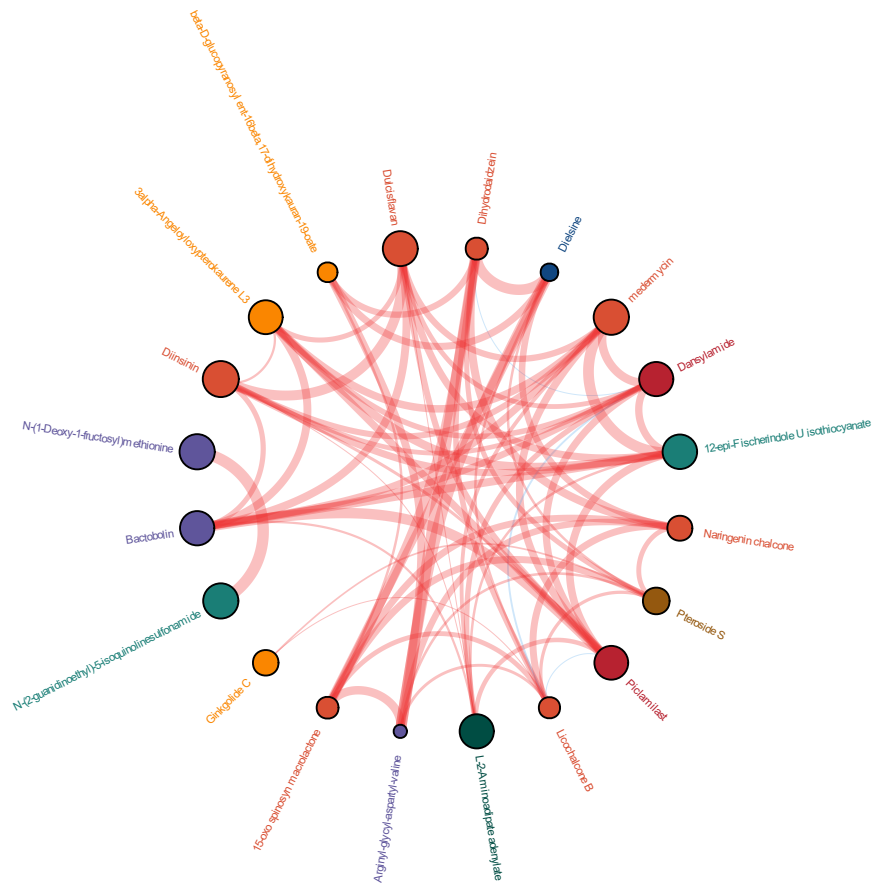

Supplement: Supplementary file 1 [file life-16-00705-s001.zip › APS,GPS/II.vs.I_pos_chord.pdf]

KEGG Classification

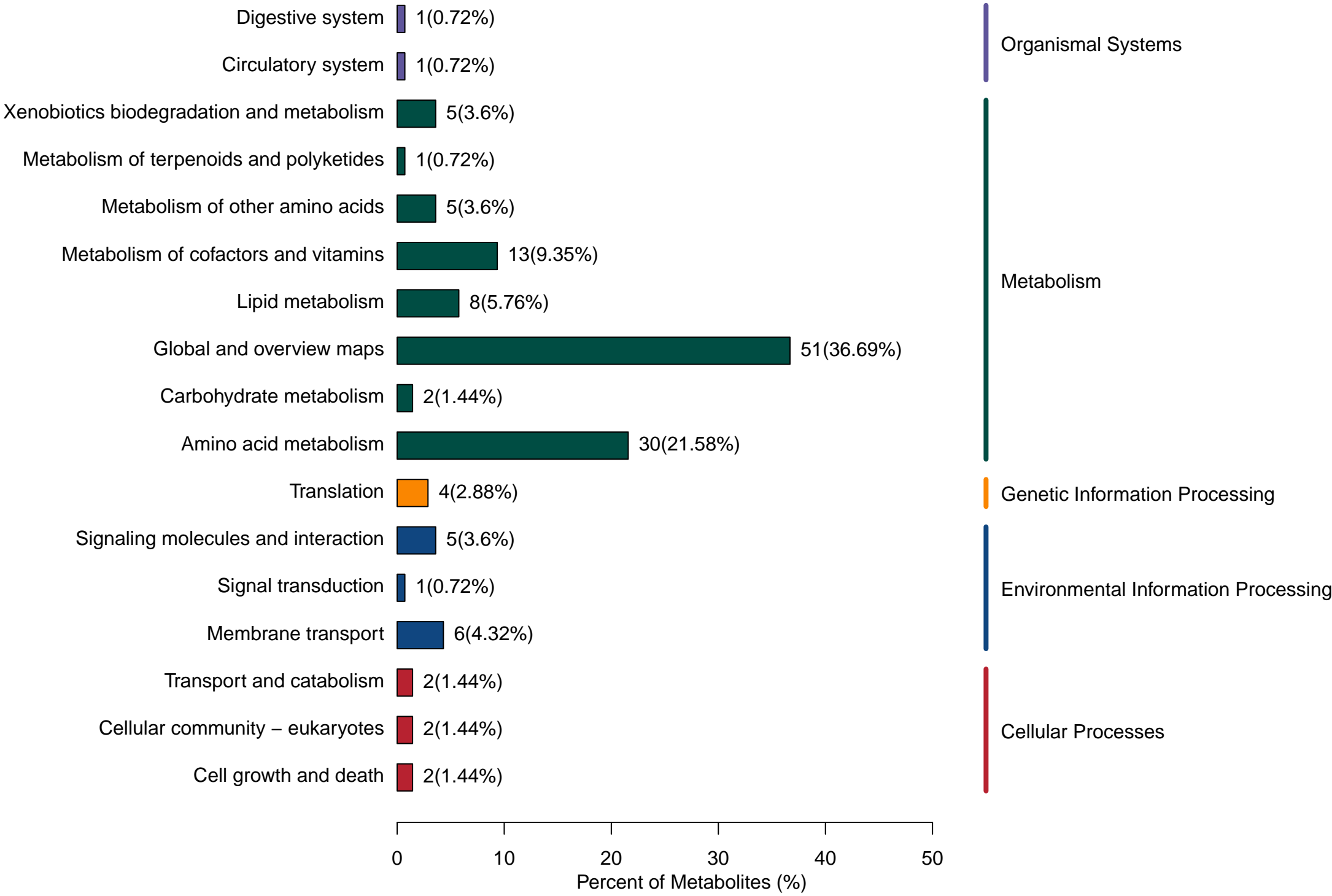

Supplement: Supplementary file 1 [file life-16-00705-s001.zip › APS,GPS/II.vs.I_pos_KEGG_classification.pdf]

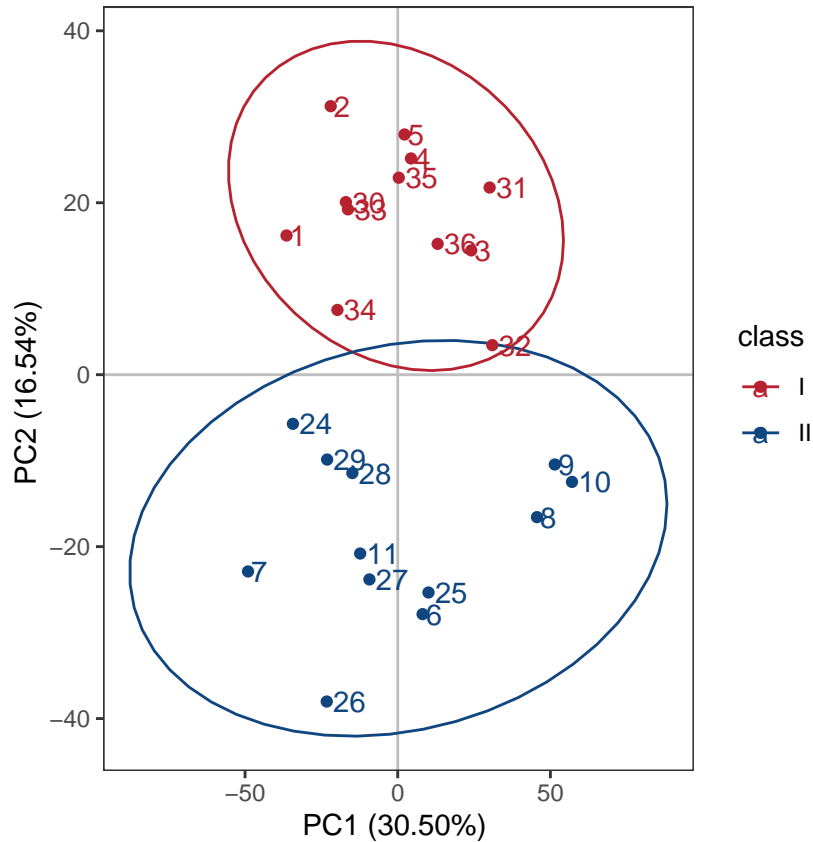

Supplement: Supplementary file 1 [file life-16-00705-s001.zip › APS,GPS/II.vs.I_pos_PCA.pdf]

class I II

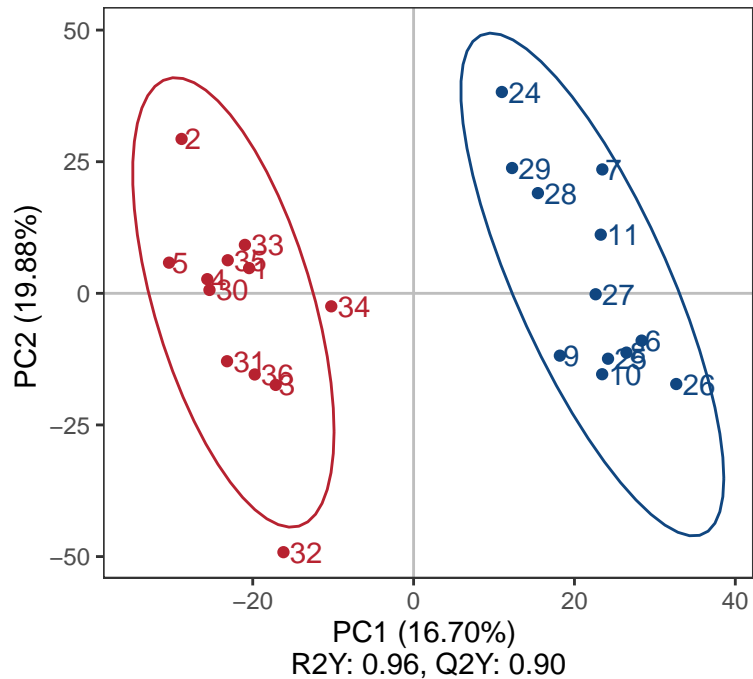

Supplement: Supplementary file 1 [file life-16-00705-s001.zip › APS,GPS/II.vs.I_pos_PLSDA-score.pdf]

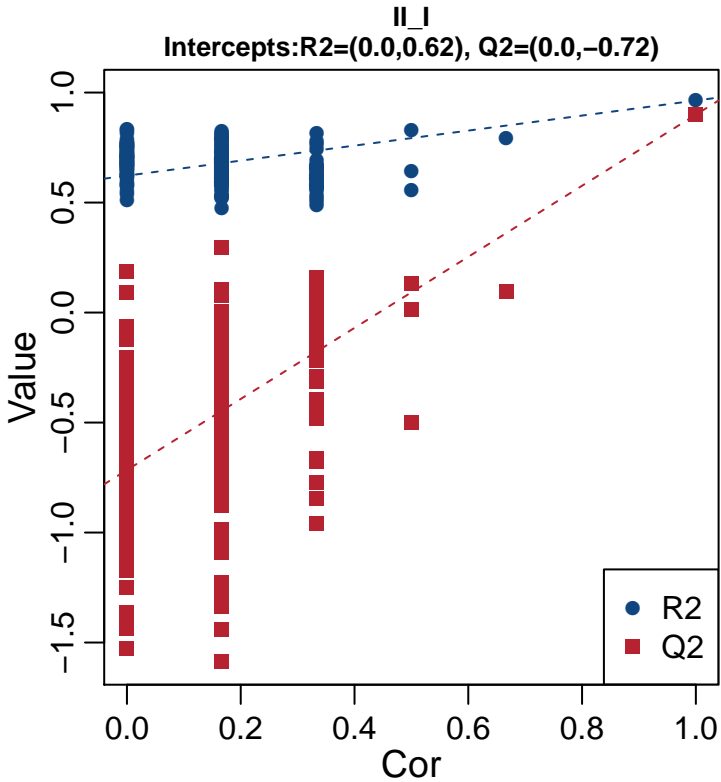

Supplement: Supplementary file 1 [file life-16-00705-s001.zip › APS,GPS/II.vs.I_pos_PLSDA-valid.pdf]

# III.vs.I\_neg

Terms

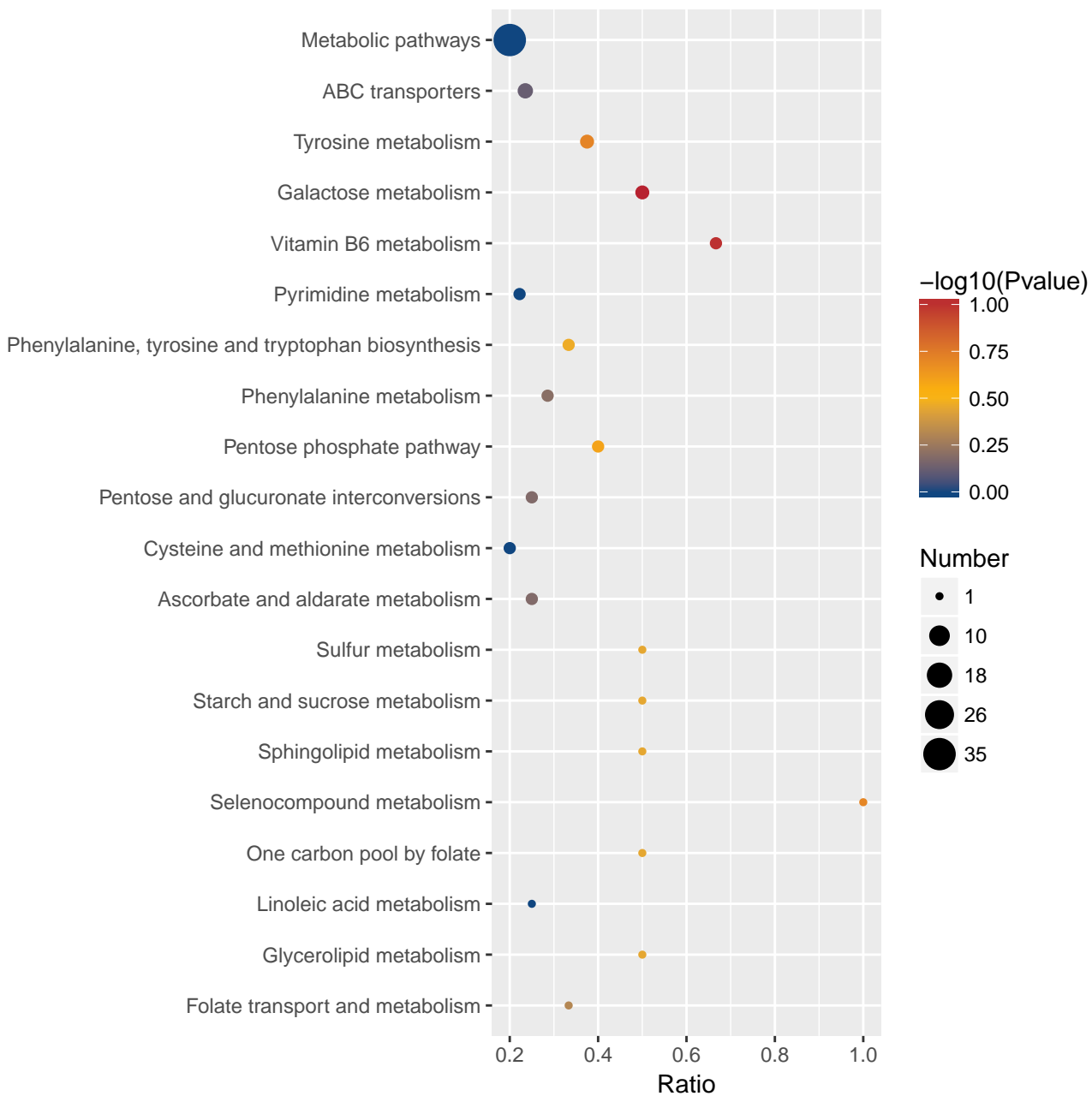

Supplement: Supplementary file 1 [file life-16-00705-s001.zip › APS,GPS/III.vs.I_neg.KEGG_Enrich.scatterplot.pdf]

III.vs.I

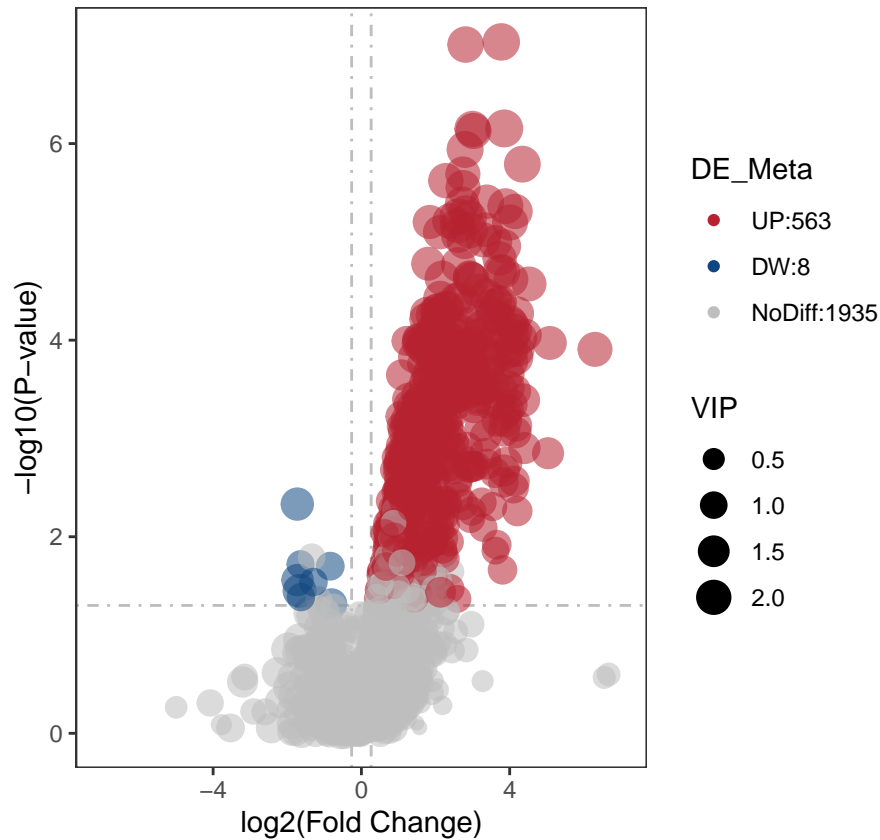

Supplement: Supplementary file 1 [file life-16-00705-s001.zip › APS,GPS/III.vs.I_neg.xls.volcano.pdf]

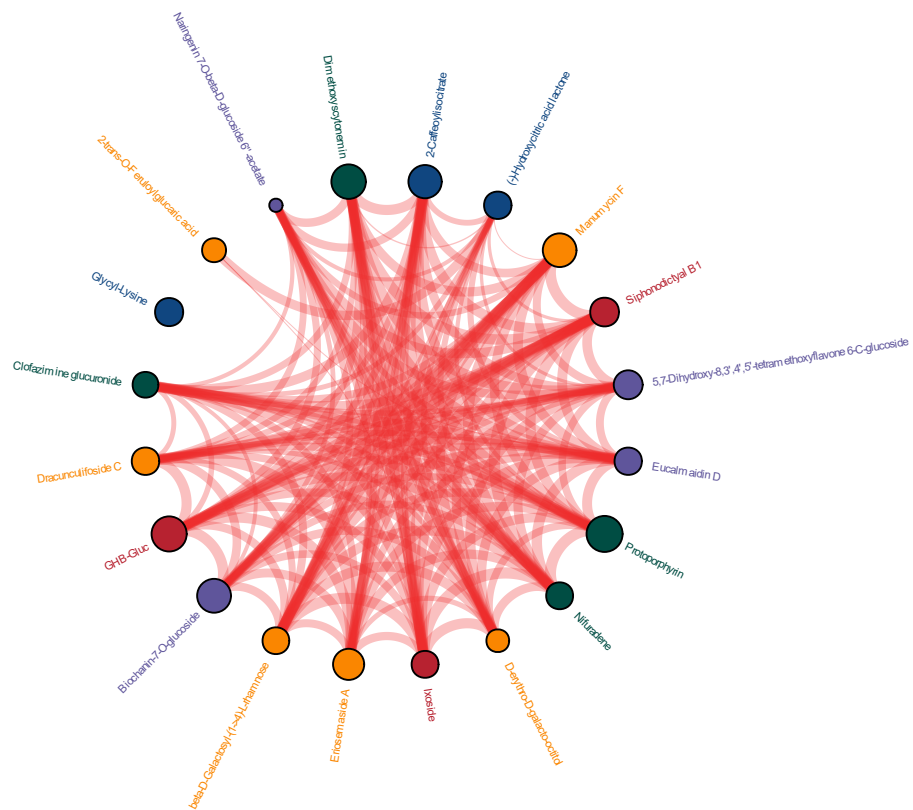

Supplement: Supplementary file 1 [file life-16-00705-s001.zip › APS,GPS/III.vs.I_neg_chord.pdf]

KEGG Classification

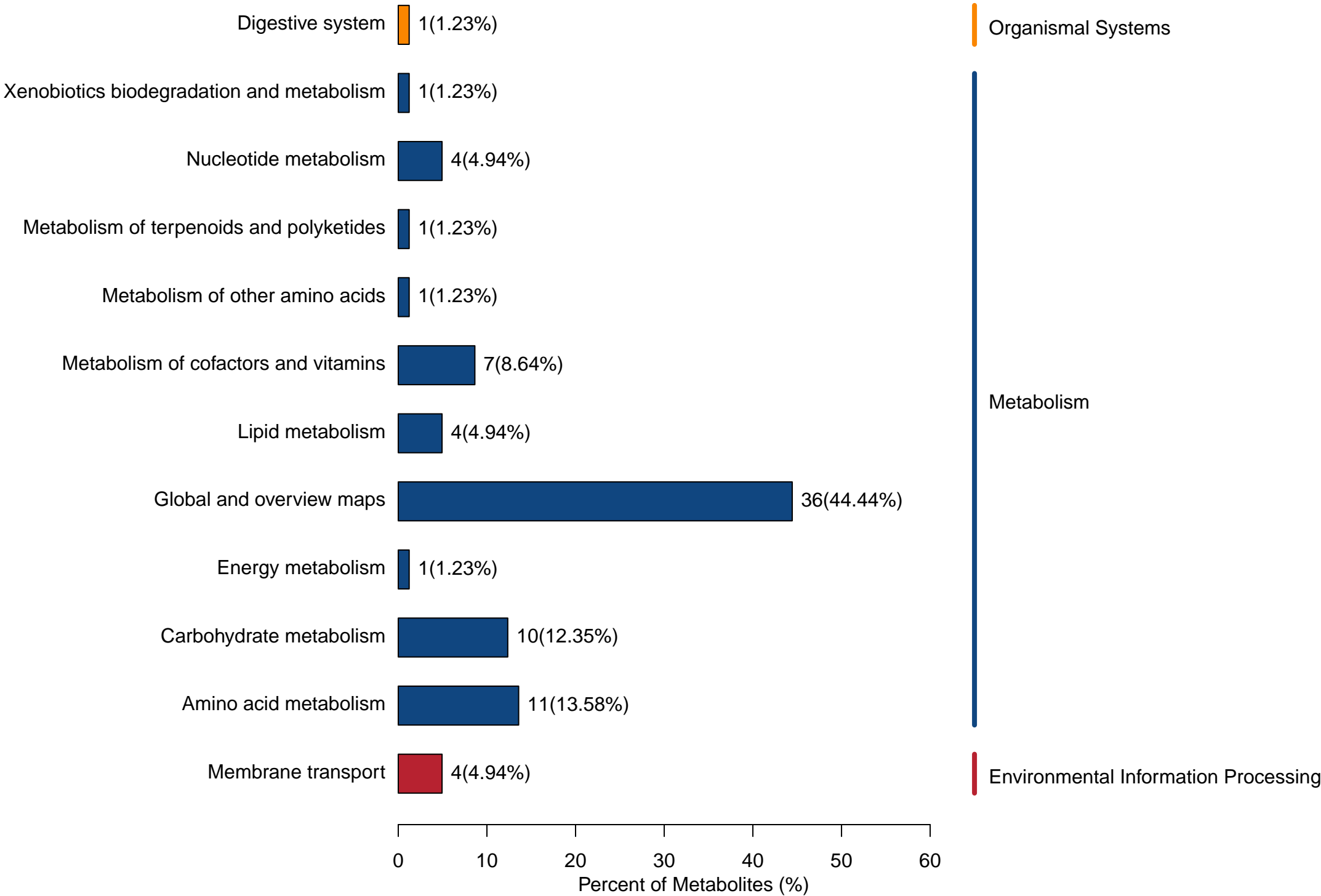

Supplement: Supplementary file 1 [file life-16-00705-s001.zip › APS,GPS/III.vs.I_neg_KEGG_classification.pdf]

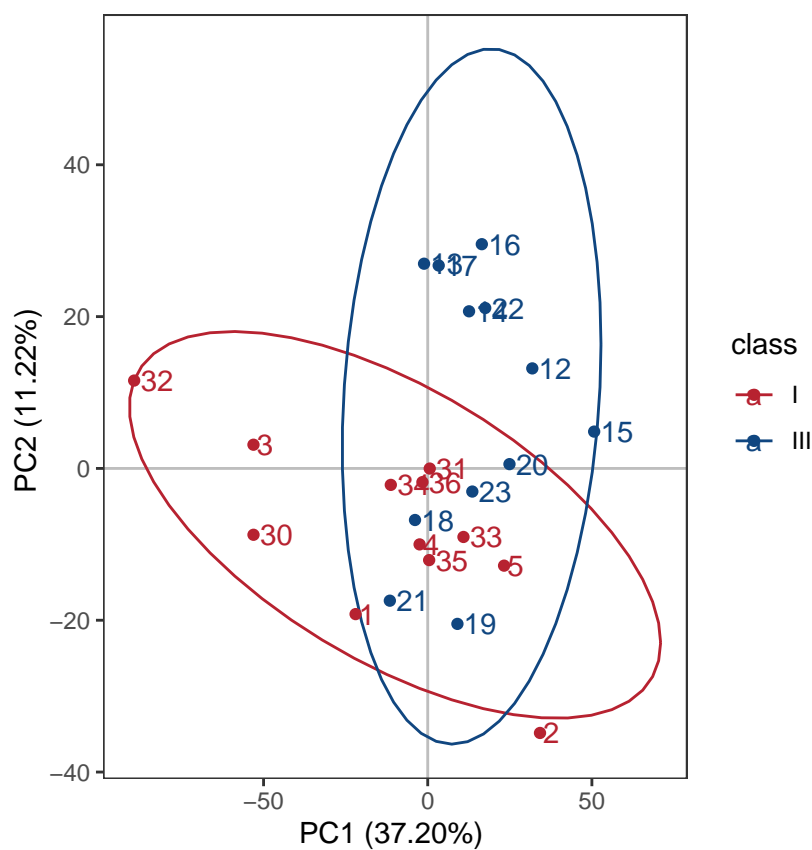

Supplement: Supplementary file 1 [file life-16-00705-s001.zip › APS,GPS/III.vs.I_neg_PCA.pdf]

class I III

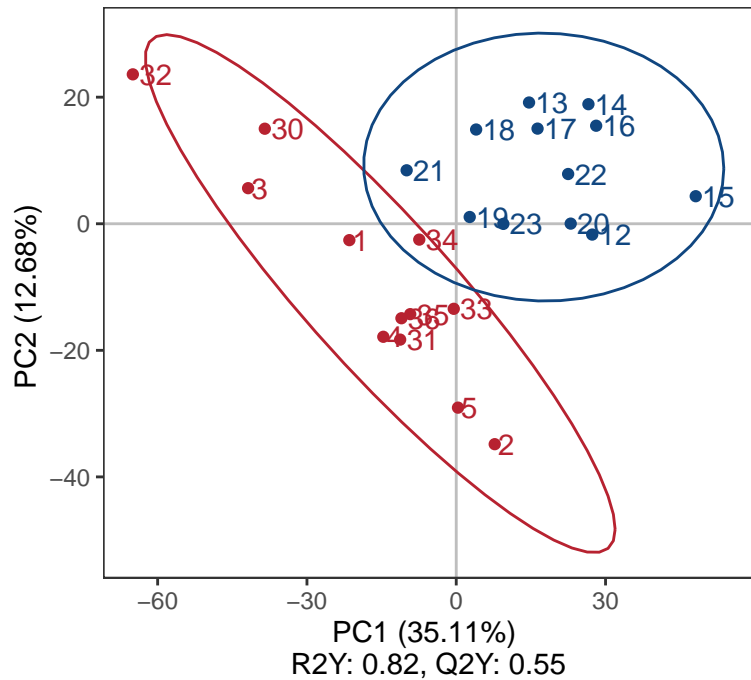

Supplement: Supplementary file 1 [file life-16-00705-s001.zip › APS,GPS/III.vs.I_neg_PLSDA-score.pdf]

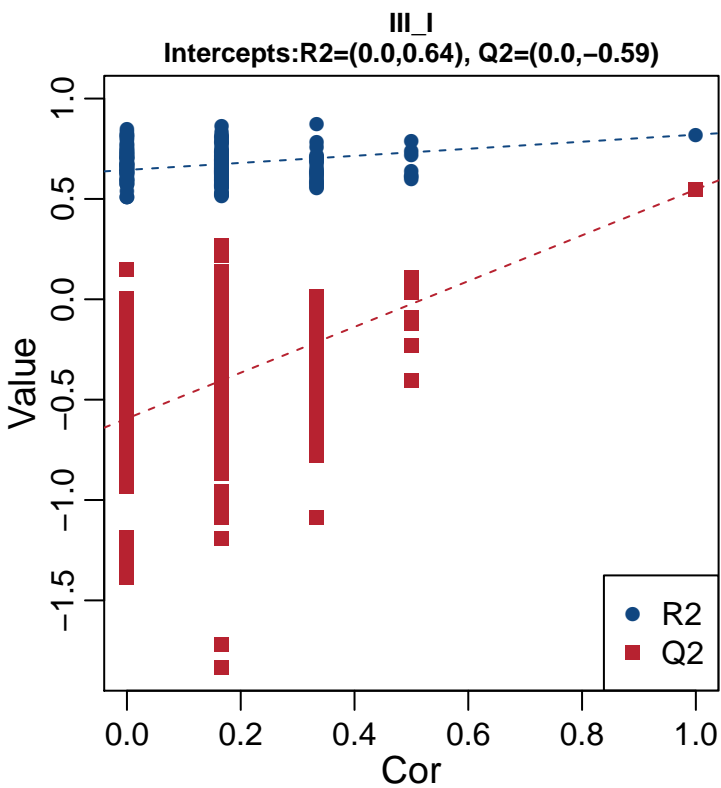

Supplement: Supplementary file 1 [file life-16-00705-s001.zip › APS,GPS/III.vs.I_neg_PLSDA-valid.pdf]

III.vs.I\_pos

Terms

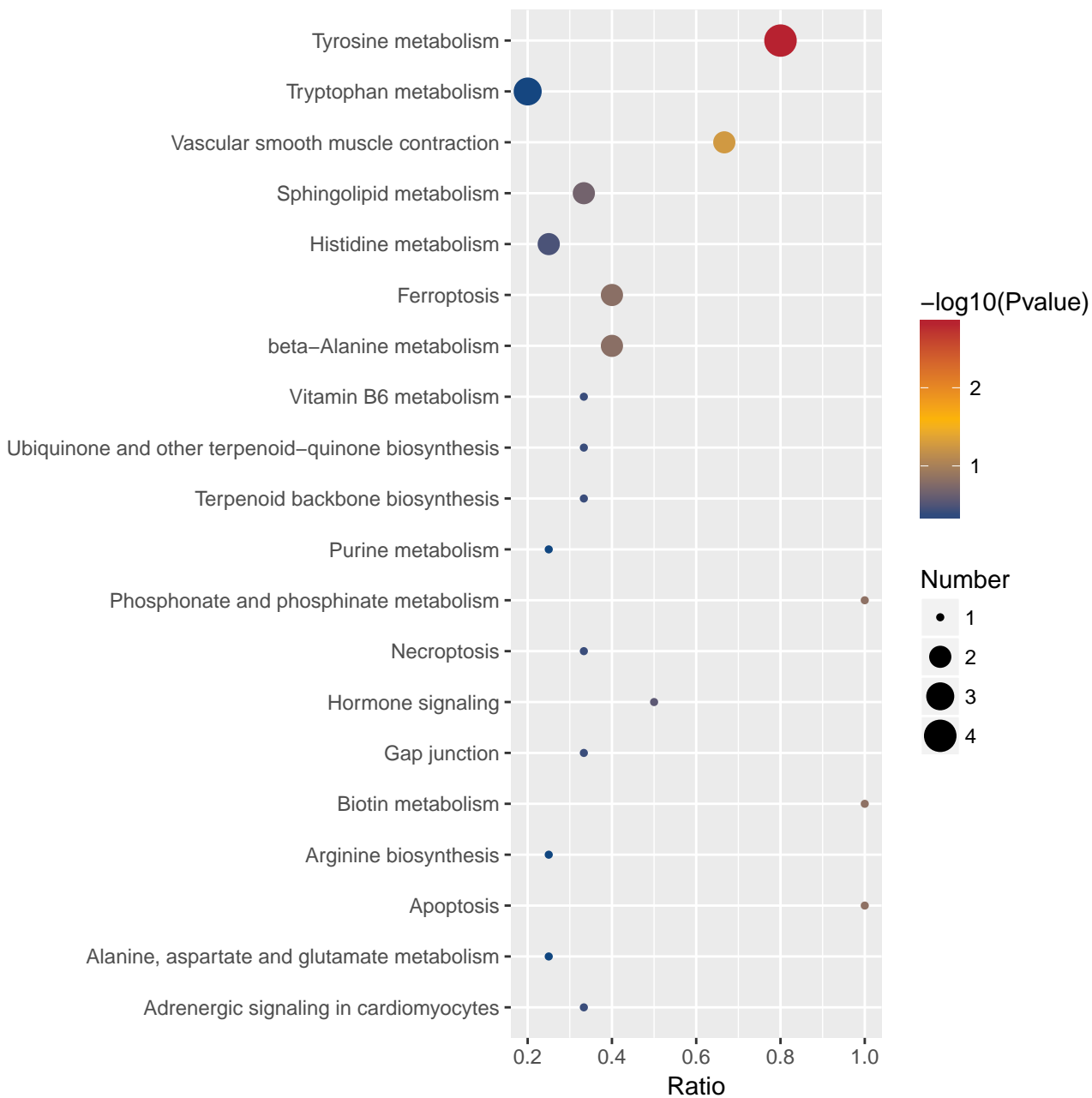

Supplement: Supplementary file 1 [file life-16-00705-s001.zip › APS,GPS/III.vs.I_pos.KEGG_Enrich.scatterplot.pdf]

III.vs.I

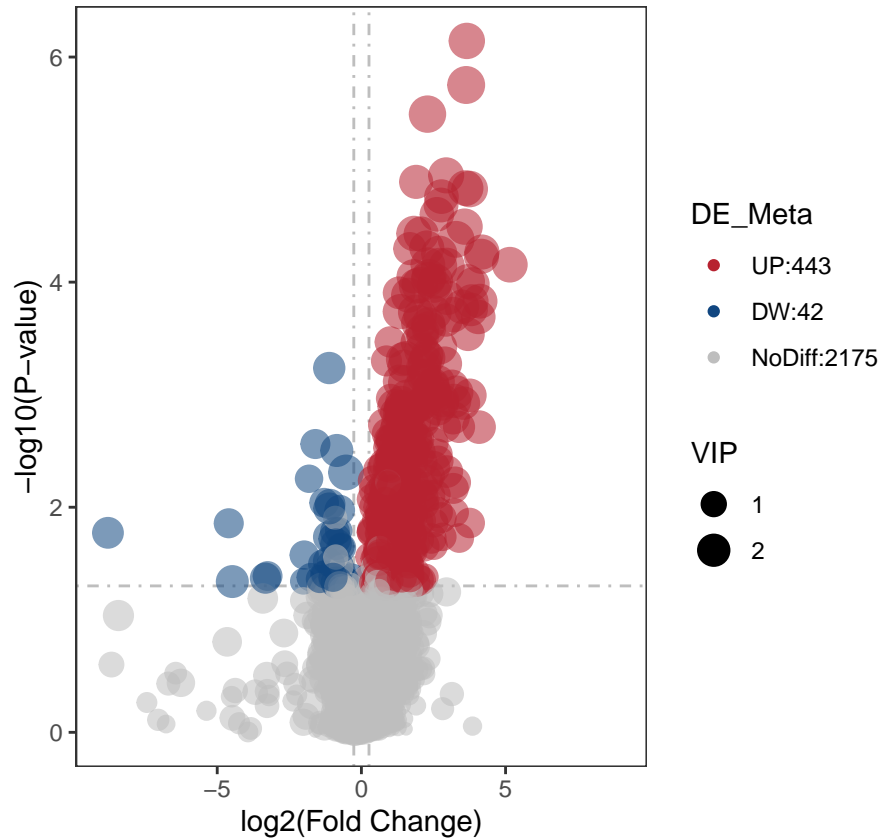

Supplement: Supplementary file 1 [file life-16-00705-s001.zip › APS,GPS/III.vs.I_pos.xls.volcano.pdf]

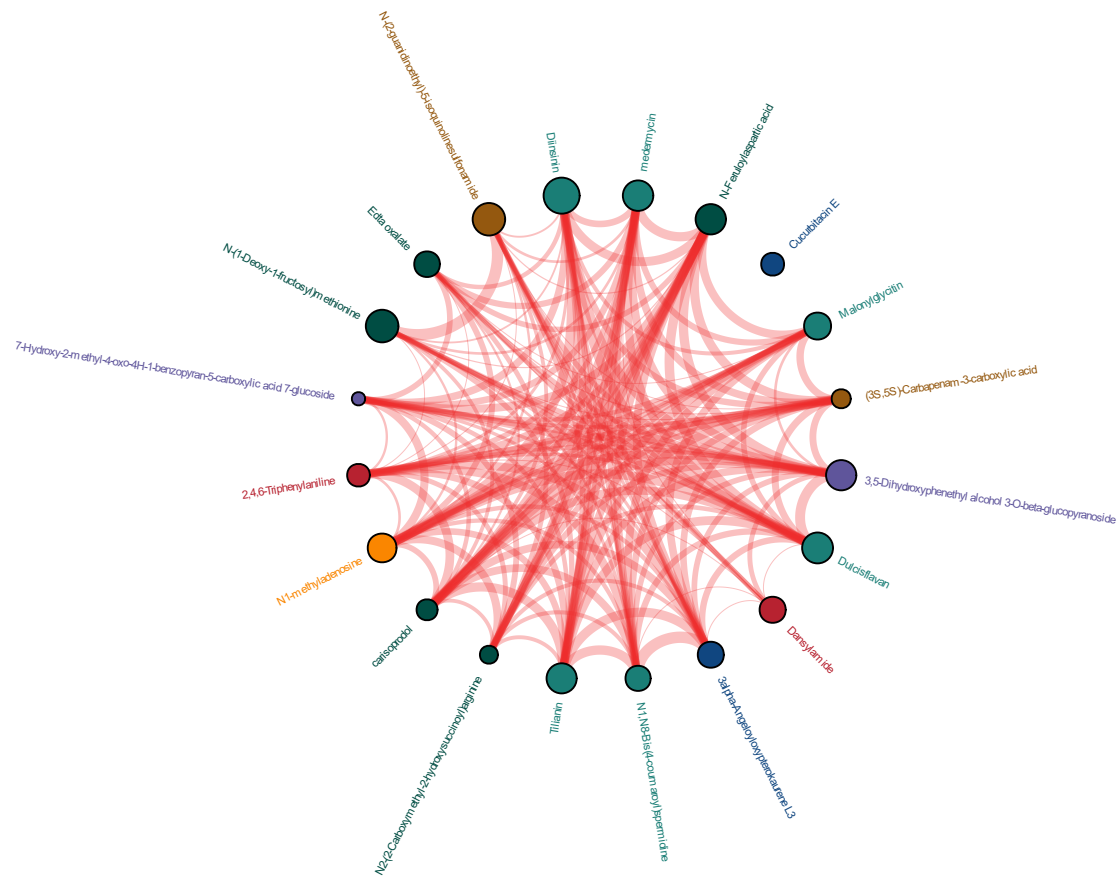

Supplement: Supplementary file 1 [file life-16-00705-s001.zip › APS,GPS/III.vs.I_pos_chord.pdf]

KEGG Classification

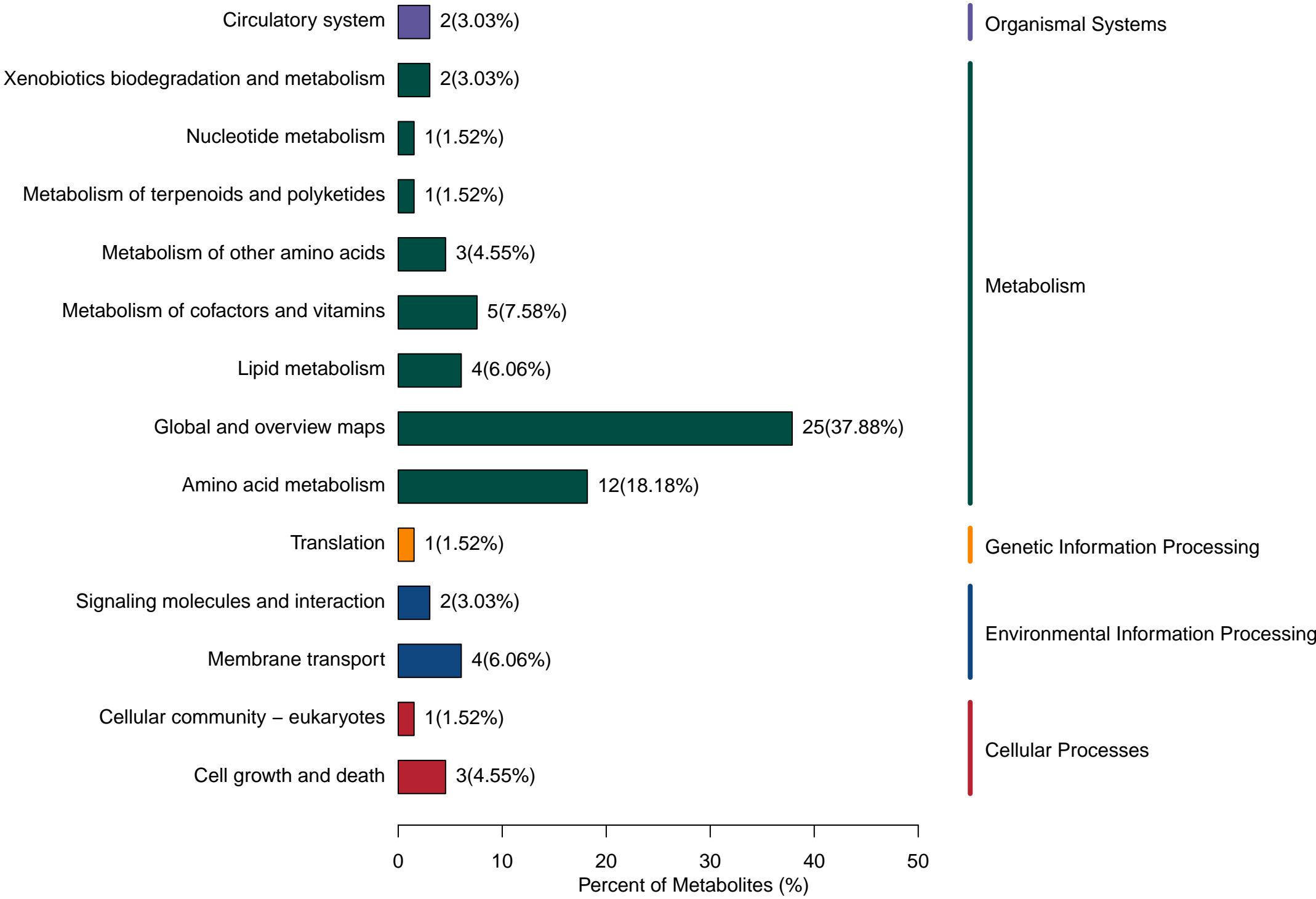

Supplement: Supplementary file 1 [file life-16-00705-s001.zip › APS,GPS/III.vs.I_pos_KEGG_classification.pdf]

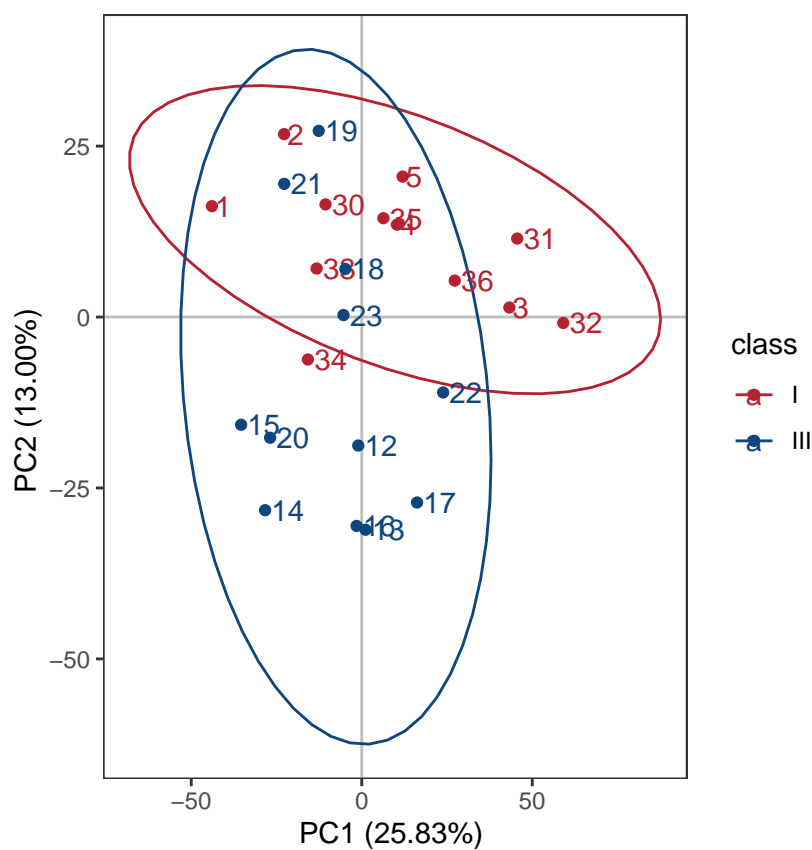

Supplement: Supplementary file 1 [file life-16-00705-s001.zip › APS,GPS/III.vs.I_pos_PCA.pdf]

class I III

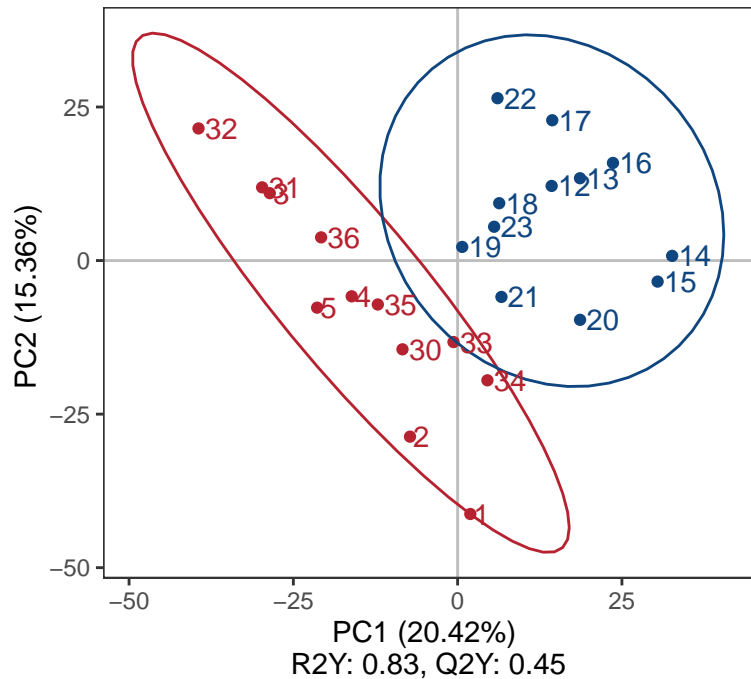

Supplement: Supplementary file 1 [file life-16-00705-s001.zip › APS,GPS/III.vs.I_pos_PLSDA-score.pdf]

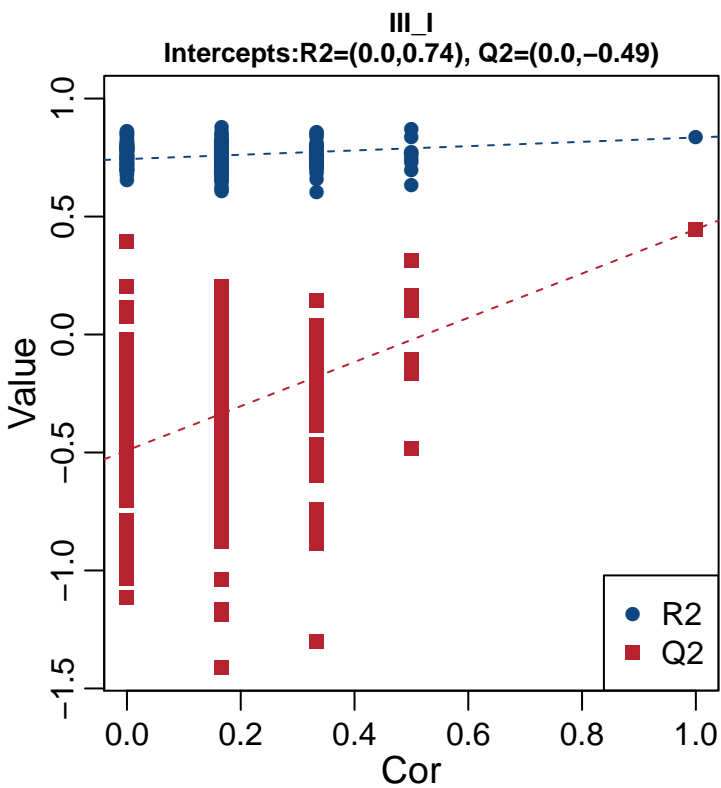

Supplement: Supplementary file 1 [file life-16-00705-s001.zip › APS,GPS/III.vs.I_pos_PLSDA-valid.pdf]
