# Supplementary material for: Effects of Plant Polysaccharides on Meat Quality of Squabs Based on Ileal Metabolomics
Source: Life (Basel). 2026 Apr 22;16(5):705. doi: 10.3390/life16050705 (PMC13208362; doi:10.3390/life16050705)
Supplement: Supplementary file 1 [file II.vs.I_neg_chord.pdf › APS,GPS/II.vs.I_neg_chord.pdf]

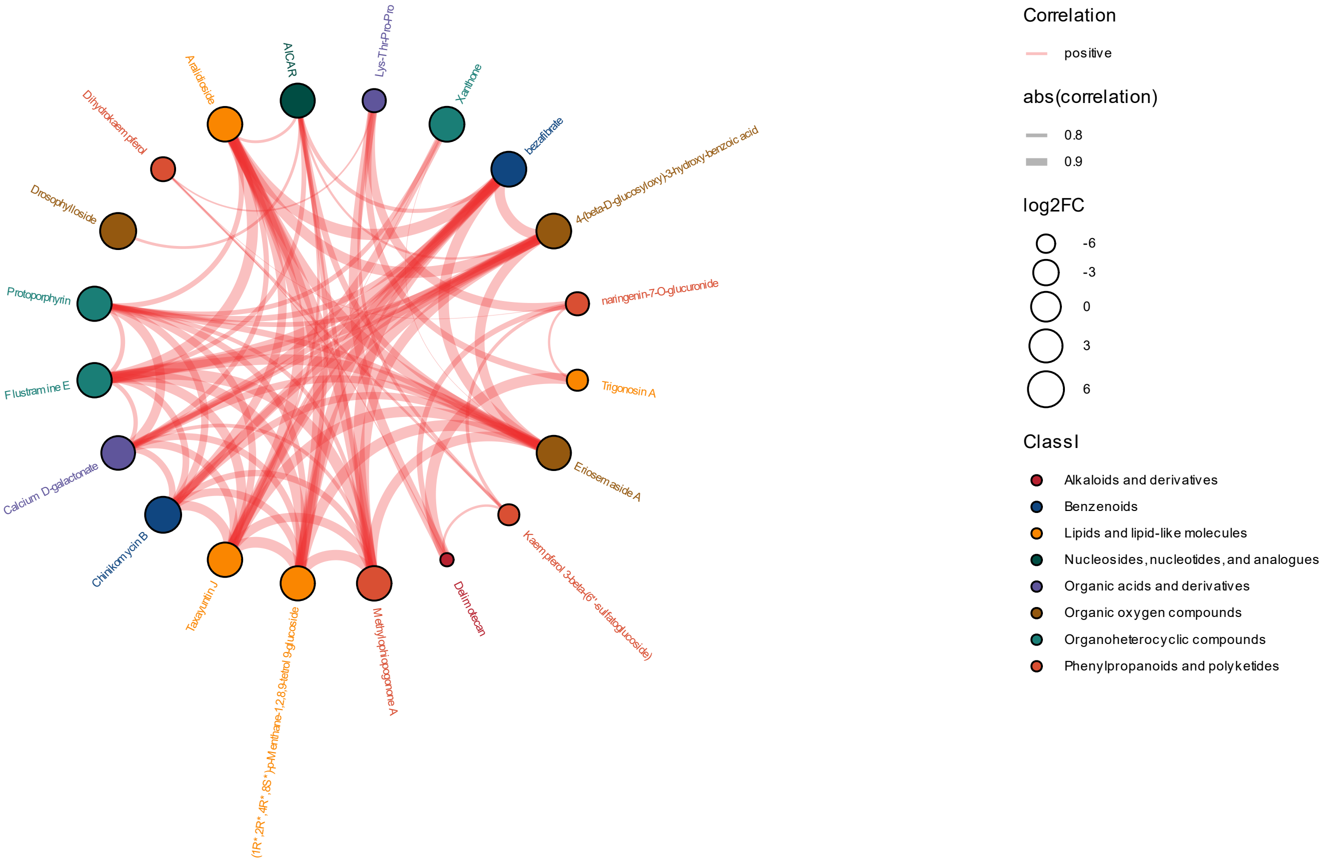

AICAR
Lys-Thr-Pro-Pro
Xanthone
bezafibrate
4-(beta-D-glucosyloxy)-3-hydroxy-benzoic acid
naringenin-7-O-glucuronide
Trigonosin A
Eriosemaside A
Kaempferol 3-beta-(6''-sulfatoglucoside)
Delimotecan
Methylophiopogonone A
(1R*,2R*,4R*,8S*)-p-Menthane-1,2,8,9-tetrol 9-glucoside
Taraxyunin J
Chinikomycin B
Calcium D-galactonate
Flustramine E
Protoporphyrin
Drosophylloside
Dihydrokaempferol
Aralidioside
Correlation
positive
abs(correlation)
0.8
0.9
log2FC
-6
-3
0
3
6
ClassI
Alkaloids and derivatives
Benzenoids
Lipids and lipid-like molecules
Nucleosides, nucleotides, and analogues
Organic acids and derivatives
Organic oxygen compounds
Organoheterocyclic compounds
Phenylpropanoids and polyketides
